# Clustering of diet, physical activity and sedentary behaviour and related physical and mental health outcomes: a systematic review

**DOI:** 10.1186/s12889-023-16372-6

**Published:** 2023-08-18

**Authors:** Noura Alosaimi, Lauren B. Sherar, Paula Griffiths, Natalie Pearson

**Affiliations:** 1 School of Sport, Exercise & Health Sciences, Loughborough University, Epinal Way, Loughborough, LE11 3TU Leicestershire UK; 2 National Institute for Health Research (NIHR) Leicester Biomedical Research Centre, Leicester, UK

**Keywords:** Clustering, Physical activity, Sedentary behaviours, Diet, Obesity, Depression, Young people

## Abstract

**Background:**

Physical activity (PA), sedentary behaviour (SB) and diet play an important role in the physical and mental health of young people. Understanding how these behaviours cluster, and the impact of clusters on health is important for the development of public health interventions. This review examines the prevalence of clusters of PA, sedentary time, and dietary behaviours, and how clusters relate to physical and mental health indicators among children, adolescents and young adults.

**Methods:**

Electronic (PubMed, Web of Science and Scopus) and manual searches were conducted for articles that were (i) observational studies including children, adolescents and/or young adults aged 5–24 years, (ii) examined the 'patterning', ‘clustering’, or ‘co-existence’ of each of PA, dietary behaviour and SB, and (iii) published in English up to and including July 2022. In addition to information on clustering, data on physical and mental health outcomes were extracted where reported. Included studies were assessed using the Cochrane risk of bias for observational studies. A narrative synthesis was conducted due to high heterogeneity. This review was registered with PROSPERO (CRD42021230976).

**Results:**

Forty-nine cross-sectional studies and four prospective cohort studies from eighteen countries reporting data from 778,415 individual participants were included. A broad range of clusters (*n* = 172) were found (healthy, unhealthy, and mixed). Mixed clusters were common (*n* = 98), and clusters of high diet quality, low PA and high SB were more prevalent in girls, while mixed clusters of high PA, high SB and low diet quality were more prevalent in boys. Unhealthy clusters comprising low moderate to vigorous PA, low consumption of fruits and vegetables, and high screen time were prevalent, particularly in those from lower socioeconomic status families. Compared to those with healthy behavioural clusters, those with unhealthy and mixed clusters had a higher adiposity, higher risk of cardiovascular disease, poorer mental health scores, and lower cardiorespiratory fitness.

**Conclusions:**

PA, SB and diet cluster in healthy, unhealthy and mixed patterns in young people that differ across sociodemographic characteristics. Unhealthy clusters are associated with poorer health outcomes. Intervention strategies targeting un-clustering multiple unhealthy behaviours should be developed and evaluated for their impact on health outcomes.

**Supplementary Information:**

The online version contains supplementary material available at 10.1186/s12889-023-16372-6.

## Background

Overweight and obesity, and their associated comorbidities, are increasing globally [1]. In the UK, the prevalence of obesity in adults has tripled over the last 20 years and continues to rise albeit at slower rates [2]. A similar pattern is seen in children (aged 10–11 years) in the UK, with obesity levels increasing from 21.0% in 2019–20 to 25.5% in 2020–21 [3]. Living with overweight or obesity is associated with long term health conditions including cardiovascular disease, diabetes, depression and premature mortality [4]. Furthermore, obesity is challenging to manage, and intervention strategies targeted at younger age groups should be a priority [5]. Modifiable health behaviours, including physical inactivity, sedentary behaviours (SB), and unhealthy dietary habits, have all been linked independently to increased risk of obesity in children, adolescents, and young adults [6–8].

Physical activity (PA), SBs, and dietary habits are developed early in life and have been shown to track through childhood [9–15] into adulthood [16]. Evidence suggests that poor lifestyle behaviours are likely to co-occur or ‘cluster’ within groups of individuals [14, 15, 17, 18], and that clustering of unhealthy behaviours increases significantly with age [19]. A recent study found that the prevalence of clustering of unhealthy behaviours increased from 29.0% in children aged 2–5 years to 73.9% among older adolescents aged 16–19 years [19]. Moreover, the most common health behaviour combinations at both time points were high screen time and unhealthy diet (the prevalence increased from 14.4% at ages 2–5 years to 45.3% for ages 16–19 years) [19].

It has been found that the clustering of unhealthy behaviours has synergistic effects on health outcomes, meaning that a combination of health behaviours is more harmful to health than the sum of the effects of each individual health behaviour; this, in turn, will increase the risk of chronic illnesses and premature mortality [20]. For example, clustering of multiple unhealthy behaviours (i.e., SB, lack of PA, and poor dietary habits) has been shown to be associated with poor mental and physical health outcomes such as increased likelihood of developing depression [21–23], anxiety, psychological distress, and weight gain [24]. Furthermore, Nelson et al. found that 51% of boys and 43% of girls had three or more behavioural risk factors that were associated with obesity, including low PA, high screen time, low consumption of fruits and vegetables (FV), and high consumption of soft drinks and snacks [25]. Conversely, clustering of beneficial health-related behaviours (being physically active, a non-smoker, moderate drinker and consuming five or more servings of FV a day) has been shown to be associated with improved mental health, improved self-reported physical health, and healthier body weight in adults [26].

Previous systematic reviews have focused on individual health behaviours (i.e. diet, PA, or SB), examined specific health outcomes or combinations of health behaviours (for example, Leech et al. focused only on weight related outcomes), and/or have focused on a narrow age group [27–36], which limits their ability to address the impact of simultaneous health behaviours on the health of young people more generally. Previous reviews have also combined or synthesised health behaviours under general/broad categories (i.e., total PA, SB or diet) rather than specifying the exact behaviour (such as moderate to vigorous physical activity (MVPA) or FV consumption), which reduces the accuracy of defining specific clusters and trends [37, 38]. Furthermore, many previous reviews have included studies in which additional health behaviours (e.g., smoking, alcohol use) have been included in the creation of clusters that also include PA, SB and dietary behaviours. Being able to disentangle these behaviours from PA, SB and diet is important for our understanding of how PA, SB and diet cluster as lifestyle behaviours, and the impact of clusters of these specific behaviours on health is important for the development of public health interventions. Despite this, clustering of these specific behaviours and their associations with both physical and mental health have not been synthesised in previous systematic reviews. Therefore, it is valuable and timely to investigate the prevalence of clustering patterns of PA, SB, and diet and their associations with physical and mental health indicators in children, adolescents, and young adults. Thus, the aim of this systematic review is to synthesize evidence from longitudinal, cross-sectional and cohort studies on the prevalence of PA, SB, and dietary behaviours clusters by age, sex, and socioeconomic status and their associations with physical and mental health in children, adolescents, and young adults.

## Methods

This review was conducted in July 2022 following the Preferred Reporting Items for Systematic Reviews and Meta-Analysis (PRISMA) [39], and was registered with the International Prospective Register of Systematic Reviews (PROSPERO) (CRD42021230976).

### Search strategy

The search strategy was developed using the Population Exposure Context Outcome (PECO) concepts, and searches were built around each concept: Population (children, adolescents and young adults), Exposure (clusters of dietary habits, PA and SB), and health Outcomes (physical and mental). Scoping searches were conducted to refine the search strategy and checked by an information specialist and the review team, ensuring that relevant studies were identified with the search syntax. Comprehensive lists of keywords were used to ensure a broad and comprehensive search (please see Additional file 1 for the full search strategy). PubMed (Medline), Web of Science and Scopus electronic databases were searched for articles up to and including July 2022. Electronic searches were supplemented by examination of the bibliographies of included studies and relevant reviews, as well as consultation with subject experts.

### Inclusion and exclusion criteria

For studies to be included they were required to: (1) be an observational study including school-aged children and/or adolescents (ages 5–19 years) and/or young adults (ages 19–24 years) as participants; (2) include an examination of the 'patterning', ‘clustering’, or ‘co-existence’ of at least one domain each of PA, dietary behaviour and SB; and (3) be published in English up to and including 24th July 2022. Studies that included all three behaviours but did not attempt to identify clusters of these three behaviours or examine an interaction or association between these behaviours were excluded. Similarly, studies that included PA, SB and dietary variables in addition to other health behaviours (e.g., alcohol consumption, sleep) were excluded if data on the three behaviours of interest could not be extracted. In addition, randomised controlled trials or any intervention studies where behaviours had been manipulated were excluded.

### Identification of relevant studies

Covidence review management software (www.covidence.org) was used for the screening and selection of records retrieved from the database and manual searches, including the removal of duplicates. Screening by title and abstract was conducted initially. A full text copy of all articles meeting the initial screening was obtained for examination. All screening was conducted by two independent reviewers, with a third reviewer assessing a random sample of 10% of the excluded studies at both title/abstract and full text stages. Any disagreements, at any stage, were resolved via consulting a third reviewer.

### Data extraction

A data extraction form was developed in Excel for the purpose of this review and was used to collate the data. The data extraction form was piloted by two reviewers on a proportion of the included studies to assess its suitability. After consultation with the review team, it was modified accordingly. The following data was extracted from each paper: (1) general information (study ID, title, author/s, date, study location (country), study aim, study type); (2) participant characteristics (participant selection and sample size, etc.); and (3) type of study, duration of study, methods and measures of health behaviours, analytical methods for clustering and statistical analyses. In addition to information on prevalence of clustering, data on any reported physical and mental health outcomes were extracted. Data extraction was performed by one reviewer and 50% of articles were checked for completeness, accuracy and consistency by a second independent reviewer. Any disagreements were resolved via discussion between the reviewers and lessons learned applied to the remaining studies.

### Risk of bias assessment

A risk of bias assessment was carried out for each of the included studies, as described in the Cochrane Handbook [40] and elsewhere [41].As this is a review of observational studies, the risk of bias assessment assessed each study against the following domains: (1) selection bias, (2) performance bias, (3) detection bias, (4) attrition bias, (5) selective reporting bias, and (6) other factors that may increase the risk of bias. Risk of bias assessments were completed independently by two reviewers, and discrepancies (*n* = 2) were resolved through discussion and the judgement of a third author. Each study was classified as either a low risk of bias, high risk of bias, or an unclear risk.

### Synthesis of results

Each included article had to include all three health behaviours of interest. For synthesis, we report the cluster names and descriptions exactly as they are reported in the original manuscripts. Results were synthesised narratively because a meta-analysis was not feasible due to the considerable heterogeneity in terms of methodological, statistical, and clinical aspects.

## Results

### Search results

**Fig. 1:**
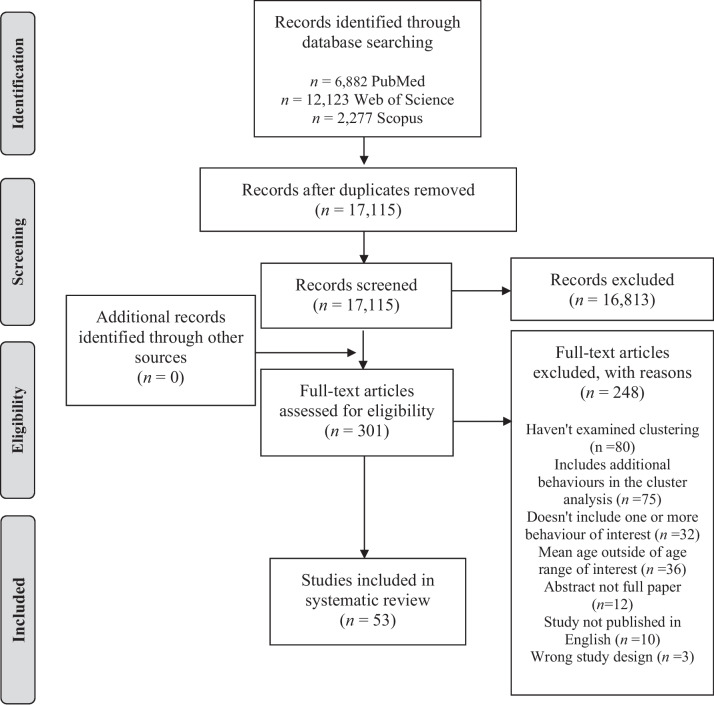
PRISMA flow diagram for the identification, screening, eligibility and inclusion of studies

A full summary of the search results is presented in the PRISMA flowchart diagram (Fig. 1). A total of 21,282 records were identified during the electronic database searches. After duplicates were removed, a total of 17,115 records remained. Of those, 16,814 records were deemed ineligible during the titles and abstracts screening process, 301 full-text articles were retained for further review, and 53 studies met the inclusion criteria.

### Study characteristics

Characteristics of the 53 included studies are summarised in Additional file 2. Studies were conducted across eighteen countries; the majority were from the US (*n* = 9) [19, 42–49], Brazil (*n* = 9) [50–58] and Australia (*n* = 5) [25, 59–62], and seven provided data from more than one country [63–69]. Studies were published between 2007 [46] and 2022 [57, 58], with the majority (87%) published within the last decade. Forty-nine studies employed a cross-sectional design and four used a longitudinal design [59, 70–72] with follow-up durations ranging between two [70, 71] and six [72] years post baseline. Most studies included adolescents (*n* = 31) [25, 42, 44, 46–48, 50–58, 63, 64, 66, 68, 72–83], thirteen included children (*n* = 13) [43, 49, 61, 62, 65, 67, 69, 70, 84–88], seven included both children and adolescents (*n* = 7) [19, 45, 59, 60, 71, 89, 90], one included adolescents and young adults (*n* = 1) [91], and only one study included young adults only (*n* = 1) [92]. Participants’ ages ranged from five [19, 49, 59–61, 70, 89] to 25 [92] years, with sample sizes ranging from 189 [78] to 304,779 [68], representing a total of 778,415 children, adolescents, and young adults. Forty-nine studies provided data on the prevalence of clusters of PA, SB and diet [19, 25, 42, 44–68, 71, 72, 74–92]. Twenty studies examined the associations between clusters and physical health outcomes (adiposity or cardiometabolic health) [43, 44, 51, 55, 56, 58, 59, 63–65, 67, 69–75, 89, 90], and one study examined associations with mental health outcomes [74].

### Risk of bias and quality assessment

**Fig. 2:**
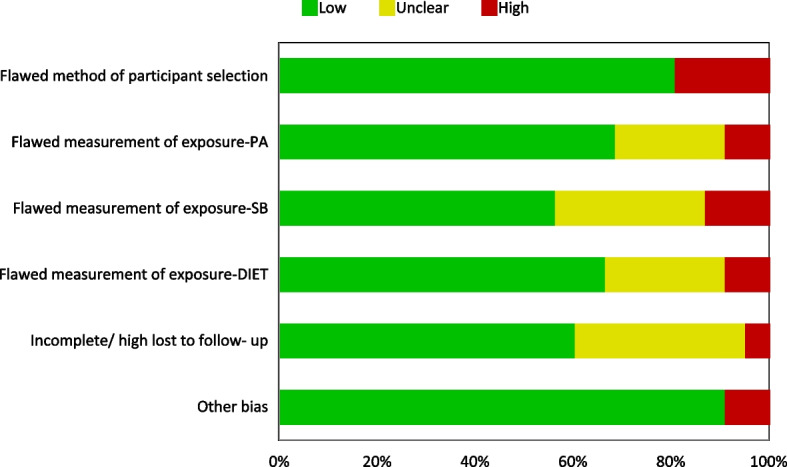
Overview of prevalence study quality and risk of bias [low, high, and unclear] assessment (*n* = 49)

**Fig. 3:**
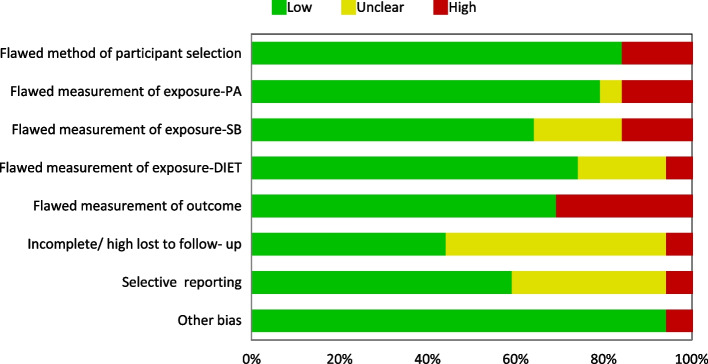
Overview of health outcomes study quality and risk of bias [low, high, and unclear] assessment (*n* = 20)

Risk of bias was conducted for all included studies. For both study types, between 5 and 30% had a high-risk judgment across all domains, while low-risk judgment varied between ~ 50–90%. Some of the domains had an unclear judgment due to lack of information (~ 5–55%) (Figs. 2 and 3).

### Overview of measures

The included studies employed a variety of methods to assess behaviours. PA and SB were measured using accelerometers in nine [44, 46, 49, 59–61, 71, 84, 87] and four [44, 59, 60, 71] studies, respectively, and were either self-reported or parent-reported in the other studies. For dietary data collection, questionnaires (e.g., a set of questions in a survey pack) were the most prevalent instrument used (*n* = 28) [45, 48, 50–54, 56–58, 61, 62, 68, 69, 71, 73, 74, 79–83, 85, 86, 88–90, 92], followed by food frequency questionnaires (FFQ) (*n* = 16) [25, 42, 43, 59, 60, 64, 65, 67, 70, 72, 75–78, 87, 91], 24-h dietary recall (*n* = 8) [19, 44, 46, 49, 55, 63, 64, 66], a diet diary (*n* = 1) [84], and multiple-pass recall methods (*n* = 1) [47].

PA was presented using varied units including daily MVPA [25, 44, 59, 60, 63, 64, 71, 87], daily PA [68, 82, 89], moderate physical activity (MPA) [44], vigorous physical activity (VPA) [44, 65],weekly MVPA [19, 43, 50, 55, 66, 74, 78, 83, 88], days per week of PA [19, 42, 51, 54, 57, 79, 80, 92], MPA [91], and VPA [48], meeting PA recommendations [46, 47, 49, 61, 62, 76, 81, 84], weekly [53, 56, 67, 70, 88, 90] or daily [45, 86] sport participation and playing outside, weekly sports at a club [69, 70, 72, 77], frequency and duration of weekly leisure time [52, 72, 75, 77], days per week of active commuting [52, 70, 81], days per week of physical education [48], and other PA/sport at school [48, 52, 70, 75, 81] and non-school [48, 73, 81], activity preferences [85], and PA score [58]. For SB, the most common outcomes measured were daily screen time (*n* = 20) [19, 25, 43, 45, 50, 51, 54–56, 58, 63, 65, 66, 71, 75, 86–90], followed by meeting screen time recommendations (*n* = 8) [46, 47, 49, 61, 62, 76, 81, 84] and daily sedentary time (*n* = 10) [44, 53, 57, 59, 60, 71, 79, 80, 82, 83]. Other studies used daily [52, 59, 64, 73, 93] or weekly [69] TV viewing, daily [42, 44, 48, 81, 92] or weekly [70] TV viewing and computer use, daily electronic media use [72, 77], and daily non screen SB (e.g. sitting and homework) [52, 63, 68, 91]. Finally, two studies used weekly screen time [74, 78], and only one used weekly sedentary time [67] and activity preference (e.g. computer use, TV viewing, reading, and tinkering) [85].

The most prevalent dietary outcomes used were daily [25, 42, 45, 51, 59, 60, 64, 68, 78–80, 82, 83, 87, 90, 91] and weekly [50, 51, 56–58, 65, 67, 69, 73, 74, 78] FV consumption, diet quality index [19, 43, 44, 63, 66, 71, 72, 77, 88], meeting dietary recommendations (i.e., FV, sugar-sweetened beverages (SSB), fat, energy dense food/drink, discretionary food, having breakfast, milk/yogurt intake, and total dietary fat and non-milk extrinsic sugar) [46, 47, 49, 61, 62, 76, 81, 84], daily [45, 64, 86, 90] and weekly [67, 69, 87] intake of SSB, daily [42, 51, 83, 90] and weekly [25, 51, 56–58, 65, 74, 81] intake of sweetened soft drinks, daily [59, 93] and weekly [58] energy dense food/drink consumption, daily [42, 45, 86, 90] and weekly [25, 56, 57, 73, 74, 81] intake of snacks, daily [42, 48, 54, 55, 75, 78, 90] and weekly [52, 53, 58, 70, 73, 75, 78, 92] consumption of diverse foods (e.g. fibre, dairy, chips, etc.), meal frequency [89], breakfast intake [45, 81, 90] or skipping [89], and daily [83, 90] and weekly [19, 81] fast-food intake. Weekly eating at restaurants [45] and food preferences [85] were each used once. All methods used to assess PA, diet, and SB are shown in Additional file 2, along with the measures of these behaviours.

### Cluster analysis methods

The included studies used various methods for clustering analysis (Additional file 2). To determine the number of meaningful clusters into which to assign participants most studies combined the Ward and k-means methods [51, 64–67, 69, 71, 77, 78, 86, 88], while one study used a combination of hierarchical method and k-means cluster analysis [63], one applied the Ward method exclusively [61], and two used a k-means non-hierarchical method [52, 74]. Latent class analysis [42, 44, 45, 56, 83, 87, 90, 91], latent profile analysis [48], K-means [73, 75] and k-medians [59, 60], principal component [70, 85], and two-step [53, 57, 58, 81, 92] cluster analyses were also used to identify subgroups of participants with similar patterns. In addition, some studies used the observed-over-expected ratio (O/E) [25, 50, 54, 68, 79, 80, 82, 84] or other descriptive analyses [19, 43, 46, 47, 49, 55, 62, 72, 76, 89] to determine the prevalence of health-related behaviour patterns observed.

### Clusters of behaviours identified

The 53 studies reviewed presented 172 unique clusters of health-related behaviours of interest, broadly classified as healthy (*n* = 28), unhealthy (*n* = 46), and mixed (*n* = 98) clusters (Additional file 3). A healthy cluster was typically characterised by good diet quality, high PA, and low SB, while an unhealthy cluster was characterised by poor diet quality, low PA and high SB. The majority fell into the mixed cluster, which included one or more healthy behaviours coexisting with one or more unhealthy behaviours (e.g. high PA, high FV, and high SB). In the healthy lifestyle clusters, only two clusters (high MVPA high FV low SSB low screen time [47, 49] and high MVPA low energy dense food/drink low SB low TV [59, 93]) were reported in two studies, while the unhealthy lifestyle cluster low MVPA low FV high screen time was reported in three studies [25, 50, 81]. Nineteen mixed lifestyle clusters were common in two studies each. Among the included studies, the health-related behaviours of interest (diet/PA/SB) were represented by a minimum of one (e.g. high PA high FV low SB) and a maximum of 13 distinct clusters.

### Prevalence of clusters of health behaviours

The prevalence of clusters of health behaviours are described in Table 1. Twenty-four healthy clusters showed a prevalence of between 0–30%, eighteen clusters between 30–60% and only one cluster between 60–100%. Mixed clusters didn’t follow a particular structure, but rather they were classified as mixed because of presence of one or more healthy behaviour coexisting with one or more unhealthy behaviour (e.g. high PA, high FV, and high SB). For the mixed clusters, ninety-six showed a prevalence between 0–30%, thirty-seven clusters between 30–60% and six clusters between 60–100%. Fifty-five unhealthy clusters showed a prevalence of between 0–30%, twenty-one clusters between 30–60% and seven clusters between 60–100%.

**Table 1:** The prevalence of clustering patterns of diet, PA and sedentary behaviours in children, adolescents, and young adults (*n* = 49)

Authors (Year)	Clusters summary and n identified	Prevalence			
		**Total sample**	**Stratified prevalence**		
			**Sex**	**SES**	**Age**
**Children (*n* = 10)**					
Bell et al., (2016) [62]	C1 ↑PA ↑FV ↓DISF ↓ST (*n* = 1)	C1 0.7%	C1 ↑ B	C1 ↑ H SES	C1 ↑ 10 years or older
Bel-Serrat et al., (2019) [65]	C1. Physically active and healthy diet ↑VPA ↑FV ↓CSD ↓ST C2. Healthy diet ↑FV ↓CSD *C1 and C2 were observed in all four groups C3. Physically active ↑VPA * Cluster 3 was observed in East Europe, South Europe/Mediterranean countries and West-Central Asia C4. Physically active and sedentary ↑VPA ↑ST * Cluster 4 only emerged in the North European countries C5. Sedentary and physically inactive ↓VPA ↑ST * North Europe, South Europe/Mediterranean countries and West-Central Asia C6. Low beverage intake, low sedentary and physically inactive ↓VPA ↓FV ↓CSD ↓ST *Cluster 6 was present in all the groups except in North Europe C7. High beverage intake and F&V intake ↑FV ↑CSD * Cluster 7 was present in both North Europe and West-Central Asia C8. Sedentary, physically inactive and healthy diet ↓VPA ↑FV ↓CSD ↑ST *Cluster 8 was emerged in North European countries C9. High beverage intake, sedentary and physically inactive ↓VPA ↑CSD ↑ST *Cluster 9 comprised in the North European and East European countries C10. Sedentary and physically active ↑VPA ↑ST *Cluster 10 was observed in East Europe only C11. High beverage intake, sedentary and physically active ↑VPA ↑CSD ↑ST *Cluster 11 was emerged in both East Europe and in South Europe/Mediterranean countries C12. Sedentary, physically active and healthy diet ↑VPA ↑FV ↓CSD ↑ST * Cluster 12 was specific to South Europe/Mediterranean countries C13. Physically active, high beverage intake, sedentary and high F&V intake ↑VPA ↑FV ↑CSD ↑ST *Cluster 13 was only observed in West-Central Asia (*n* = 13)	Total North Europe C1 (21.3%) C2 (29.7%) C4 (9.0%) C5 (14.0%) C7 (11.7%) C8 (11.1%) C9 (3.2%) East Europe C1 (23.4%) C2 (17.1%) C3 (16.3%) C6 (14.4%) C9 (6.4%) C10 (11.5%) C11 (11.0%) South Europe/Mediterranean countries C1 (13.6%) C2 (24.9%) C3 (12.8%) C5 (9.8%) C6 (19.7%) C12 (8.4%) C11 (10.8%) West-Central Asia C1 (15.0%) C2 (17.6%) C3 (12.7%) C5 (10.7%) C6 (16.1%) C7 (14.6%) C13 (13.3%)	Boys North Europe C1 (52.1%) C2 (44.1%) C4 (54.4%) C5 (50.8%) C7 (54.6%) C8 (63.8%) C9 (61.4%) East Europe C1 (48.4%) C2 (45.2%) C3 (51.8%) C6 (48.6%) C9 (50.8%) C10 (53.9%) C11 (54.8%) South Europe/Mediterranean countries C1 (52.8%) C2 (46.4%) C3 (54.5%) C5 (49.9%) C6 (47.6%) C12 (52.0%) C11 (52.9%) West-Central Asia C1 (49.0%) C2 (47.6%) C3 (52.6%) C5 (54.9%) C6 (48.0%) C7 (48.6%) C13 (53.9%) Girls North Europe C1 (47.9%) C2 (55.9%) C4 (45.6%) C5 (49.2%) C7 (45.4%) C8 (36.2%) C9 (38.6%) East Europe C1 (51.6%) C2 (57.8%) C3 (48.2%) C6 (51.4%) C9 (49.2%) C10 (46.1%) C11 (45.2%) South Europe/Mediterranean countries C1 (47.2%) C2 (53.6%) C3 (45.5%) C5 (50.1%) C6 (52.4%) C12 (48.0%) C11 (47.1%) West-Central Asia C1 (51.0%) C2 (52.4%) C3 (47.4%) C5 (45.1%) C6 (52.0%) C7 (51.4%) C13 (46.1%)	NA	NA
Cameron et al., (2011) [61]	C1. Young physical activity enthusiasts ↑MVPA C2. All-round healthy behaviors ↑FV ↓ED ↓ST C3. Screen time focused ↑MVPA ↑FV ↓ED ↑ST C4. Low on fruit and vegetable and physical activity ↓MVPA ↓FV C5. Energy dense eaters who watch ↑ED ↑ST (*n* = 5)	24.2% met the Australian guidelines 10% met none of the Australian guidelines	NA	NA	C1 ↑ younger age
Elsenburg et al., (2014) [84]	C1. Low physical activity / High screen time / Low fruit and vegetable intake / High MAR ↓MVPA ↓FV ↑MAR ↑ST C2. Low physical activity / High screen time / Low fruit and vegetable intake / Low MAR ↓MVPA ↓FV ↓MAR ↑ST C3. Low physical activity / High screen time / High fruit and vegetable intake / High MAR ↓MVPA ↑FV ↑MAR ↑ST (*n* = 3)	C1. Low physical activity / High screen time / Low fruit and vegetable intake / High MAR Observed % = 4.42 Expected % = 3.37 O/E (95% CI) = 1.31 (1.04, 1.59) C2. Low physical activity / High screen time / Low fruit and vegetable intake / Low MAR Observed % = 0.75 Expected % = 0.91 O/E (95% CI) = 0.82 (0.38, 1.27) C3. Low physical activity / High screen time / High fruit and vegetable intake / High MAR Observed % = 1.97 Expected % = 2.63 O/E (95% CI) = 0.75 (0.50, 0.99)	NA	NA	NA
Kunin-Batson et al., (2015) [49]	C1. Physical activity / Screen time / Sugar-sweetened beverage intake / Fruit and vegetable intake ↑MVPA ↑FV ↓SSB ↓ST C2. Physical activity / Screen time / Sugar-sweetened beverage intake ↑MVPA ↓SSB ↓ST C3. Physical activity / Screen time / Fruit and vegetable intake ↑MVPA ↑FV ↓ST (*n* = 3)	C1. Physical activity / Screen time / Sugar-sweetened beverage intake / Fruit and vegetable intake (2%) C2. Physical activity / Screen time / Sugar-sweetened beverage intake (6%) C3. Physical activity / Screen time / Fruit and vegetable intake (1%) Only 2% met all 4 guidelines 19% met none of the guidelines	NA	NA	NA
López-Gil et al., (2020) [88]	C1. Cluster 1 ↑MVPA ↑DQ ↑ST C2. Cluster 2 ↓MVPA ↓DQ ↑ST C3. Cluster 3 ↓MVPA ↓DQ ↓ST (*n* = 3)	C1. Cluster 1 Total (23.8%) C2. Cluster 2 Total (30.0%) C3. Cluster 3 Total (46.2%)	C1. Cluster 1 Boys (70.2%) Girls (29.8%) C2. Cluster 2 Boys (52.3%) Girls (47.7%) C3. Cluster 3 Boys (46.6%) Girls (53.4%)	NA	C2 ↑ oldest age C3 ↑ youngest age
Pereira et al., (2015) [87]	C1. Low MVPA/high FV/high screen time/high sugar drinks ↓MVPA ↑FV ↑SSB ↑ST C2. Low MVPA/high FV/high screen time/low sugar drinks ↓MVPA ↑FV ↓SSB ↑ST C3. Low MVPA/low FV/ high screen time/high sugar drinks ↓MVPA ↓FV ↑SSB ↑ST (*n* = 3)	C1. Low MVPA/high FV/high screen time/high sugar drinks fo = 10 fe = 21.44 *x*^2^ = 6.10 *p*-Value = 0.014 C2. Low MVPA/high FV/high screen time/low sugar drinks fo = 9 fe = 21.44 *x*^2^ = 7.22 *p*-Value = 0.007 C3. Low MVPA/low FV/ high screen time/high sugar drinks fo = 3 fe = 21.44 *x*^2^ = 15.86 *p*-Value = < 0.001	NA	NA	NA
Rodenburg et al., (2013) [85]	C1. Unhealthy-food-and-drink-preference ↑UHF ↓HF C2. Active-leisure-time-preferences ↑PA C3. Sedentary-sweetened-drinks-preferences ↑TVCOM ↑SSB (*n* = 3)	–-	C1 ↓ G C2 ↓ G	NA	C1 ↓ younger age
Santaliestra-Pasías et al., (2015) [67]	C1. Cluster 1 ↑PA ↓FV ↓SSB ↓SB C2. Cluster 2 ↓PA ↓FV ↓SSB ↑SB C3. Cluster 3 ↑PA ↓FV ↓SSB ↑SB C4. Cluster 4 ↓PA ↓FV ↑SSB ↓SB C5. Cluster 5 ↓PA ↓FV ↓SSB ↓SB C6. Cluster 6 ↓PA ↑FV ↓SSB ↓SB (*n* = 6)	–-	C1. Cluster 1 Boys (14%) Girls (14%) C2. Cluster 2 Boys (21%) Girls (22%) C3. Cluster 3 Boys (10%) Girls (13%) C4. Cluster 4 Boys (9%) Girls (9%) C5. Cluster 5 Boys (29%) Girls (25%) C6. Cluster 6 Boys (17%) Girls (17%)	C4 ↑ L SES	C1 and C3 ↑ older age C5 and C6 ↑ younger age
Yang-Huang et al., (2020) [86]	C1. Relatively healthy lifestyle ↑PA ↓SSB ↓SN ↓ST C2. High screen time and physically inactive ↓PA ↑ST C3. Physically active, high snacks and sugary drinks ↑PA ↑SN ↑SSB (*n* = 3)	C1. Relatively healthy lifestyle Total (35.6%) C2. High screen time and physically inactive Total (30.0%) C3. Physically active, high snacks and sugary drinks Total (34.4%)	C1. Relatively healthy lifestyle Boys (46.8%) Girls (53.2%) C2. High screen time and physically inactive Boys (51.3%) Girls (48.7%) C3. Physically active, high snacks and sugary drinks Boys (54.1%) Girls (45.9%)	C1 ↑ H SES C2 ↑ L SES L SES (maternal education) OR of 0.28 to be in C1 L SES (maternal education) OR of 1.45 to be in C2 and OR of 2.28 to be in C3 L SES (households income) OR of 0.59 to be in C1 and OR of 1.57 to be in C2	NA
**Adolescents (*n* = 30)**					
Berlin et al., (2017) [48]	C1. Sedentary + Unbalanced Diet ↓PA ↓FV ~ FF ~ ST C2. Active + Healthy Diet ↑PA ↑FV ↓FF ↓ST C3. Screen-time + Recreational Food ~ PA ~ FV ↑FF ↑ST (*n* = 3)	C1. Sedentary + Unbalanced Diet Total (48.7%) C2. Active + Healthy Diet Total (42.7%) C3. Screen-time + Recreational Food Total (8.6%)	C1. Sedentary + Unbalanced Diet Boys (47.8%) Girls (52.2%) C2. Active + Healthy Diet Boys (49.6%) Girls (50.4%) C3. Screen-time + Recreational Food Boys (68.2%) Girls (31.8%)	C1 ↑ L SES C3 ↑ L SES	NA
Cuenca-García et al., (2013) [63]	C1. Healthy diet and active ↑MVPA ↑DQ ↓ST ~ SBHW C2. Healthy diet and academic ~ MVPA ↑DQ ~ ST ↑SBHW C3. Healthy diet and inactive ↓MVPA ~ DQ ~ ST ~ SBHW C4. Unhealthy diet and screen user ~ MVPA ↓DQ ↑ST ~ SBHW C5. Unhealthy diet and active ↑MVPA ↓DQ ~ ST ~ SBHW (*n* = 5)	C1. Healthy diet and active Total (15%) C2. Healthy diet and academic Total (19%) C3. Healthy diet and inactive Total (38%) C4. Unhealthy diet and screen user Total (17%) C5. Unhealthy diet and active Total (11%)	C1. Healthy diet and active Boys (17%) Girls (13%) C2. Healthy diet and academic Boys (13%) Girls (23%) C3. Healthy diet and inactive Boys (33%) Girls (43%) C4. Unhealthy diet and screen user Boys (21%) Girls (13%) C5. Unhealthy diet and active Boys (15%) Girls (8%)	NA	C1 and C5 ↑ younger age C4 ↑ older B C2 ↑ older G
Cureau et al., (2018) [55]	C1 ↓MVPA ↓FIB ↑ST (*n* = 1)	C1 Total (29.4)	C1 Boys (23.8) Girls (35.1)	NA	NA
Dantas et al., (2018) [51]	C1. Cluster 1 ↑PA ↓FV ↓SSB ↓SB C2. Cluster 2 ↓PA ↓FV ↓SSB ↑SB C3. Cluster 3 ↑PA ↓FV ↓SSB ↑SB C4. Cluster 4 ↓PA ↓FV ↑SSB ↓SB C5. Cluster 5 ↓PA ↓FV ↓SSB ↓SB C6. Cluster 6 ↓PA ↑FV ↓SSB ↓SB (*n* = 6)	–-	C1. Cluster 1 Boys (22.0%) Girls (17.9%) C2. Cluster 2 Boys (23.1%) Girls (24.0%) C3. Cluster 3 Boys (15.1%) Girls (15.0%) C4. Cluster 4 Boys (8.6%) Girls (10.2%) C5. Cluster 5 Boys (17.2%) Girls (18.1%) C6. Cluster 6 Boys (14.0%) Girls (14.8%)	C4 ↑ L SES	C1 and C3 ↑ younger age C5 and C6 ↑ older age
de Mello et al., (2021) [56]	Whole sample C1 ↓PA ↓FV ↑SSS ↑ST C2 ↑PA ↑FV ↓SSS ↑ST Boys C1 ↓PA ↑FV ↑SSS ↑ST C2 ↑PA ↑FV ↓SSS ↑ST Girls C1 ↓PA ↑FV ↑SSS ↑ST C2 ↑PA ↓FV ↑SSS ↑ST (*n* = 2)	Whole sample C1 56.16% C2 43.84%	Boys C1 49.48% C2 50.52% Girls C1 34.74% C2 65.26%	NA	NA
de Mello et al., (2022) [57]	C1 ↑PA ↑FV ~ SB C2 ↓PA ↑UHF ↑SB C3 ↓PA ~ FV ~ SB C4 ↓PA ↓FV ↓UHF ~ SB (*n* = 4)	–-	C2, C3, and C4 ↑ G C1 ↑ B	C2, C3, and C4 in age 11–12 years maternal education was unknown C1, C2, and C4 in age 18–19 years ↓ SES	Age 11–12 years C1 (27.9%) C2 (16.5%) C3 (33.9%) C4 (21.7%) Age 13–15 years C1 (23.6%) C2 (33.9%) C3 (42.5%) Age 16–17 years C1 (28.1%) C2 (28.1%) C3 (43.8%) Age 18–19 years C1 (21.3%) C2 (43.7%) C4 (35%)
Foltz et al., (2011) [47]	C1 ↑MVPA ↑FV ↓SSB ↓ST (*n* = 1)	C1 Total (0.4%)	C1 Boys (0.5%) Girls (0.4%)	↓ SES less likely to meet all guidelines	NA
Hardy et al., (2012) [25]	C1. Low fruit /vegetable intake / Low physical activity / High screen time / High soft drink intake / High snack intake ↓MVPA ↓FV ↑SN ↑CSD ↑ST C2. Low fruit /vegetable intake / Low physical activity / High screen time / High soft drink intake ↓MVPA ↓FV ↑CSD ↑ST C3. Low fruit /vegetable intake / Low physical activity / High screen time / High snack intake ↓MVPA ↓FV ↑SN ↑ST C4. Low physical activity / High screen time / High soft drink intake / High snack intake ↓MVPA ↑SN ↑CSD ↑ST C5. Low fruit /vegetable intake / Low physical activity / High screen time ↓MVPA ↓FV ↑ST C6. Low physical activity / High screen time / High snack intake ↓MVPA ↑SN ↑ST C7. Low physical activity / High screen time / High soft drink intake ↓MVPA ↑CSD ↑ST (*n* = 7)	–-	Boys C1. Low fruit /vegetable intake / Low physical activity / High screen time / High soft drink intake / High snack intake Observed % = 6.9 Expected % = 2.7 O/E (95%CI) = 2.6 (1.9 – 3.6) C2. Low fruit /vegetable intake / Low physical activity / High screen time / High soft drink intake Observed % = 4.2 Expected % = 3.3 O/E (95%CI) = 1.3 (0.8 – 2.0) C3. Low fruit /vegetable intake / Low physical activity / High screen time / High snack intake Observed % = 3.8 Expected % = 5.3 O/E (95%CI) = 0.7 (0.5 – 1.0) C4. Low physical activity / High screen time / High soft drink intake / High snack intake Observed % = 4.2 Expected % = 3.1 O/E (95%CI) = 1.4 (0.7 – 2.5) C5. Low fruit /vegetable intake / Low physical activity / High screen time Observed % = 7.2 Expected % = 6.5 O/E (95%CI) = 1.1 (0.8 – 1.6) C6. Low physical activity / High screen time / High snack intake Observed % = 4.9 Expected % = 6.2 O/E (95%CI) = 0.8 (0.5 – 1.2) C7. Low physical activity / High screen time / High soft drink intake Observed % = 2.5 Expected % = 3.8 O/E (95%CI) = 0.7 (0.4 – 1.1) Girls C1. Low fruit /vegetable intake / Low physical activity / High screen time / High soft drink intake / High snack intake Observed % = 3.7 Expected % = 1.4 O/E (95%CI) = 2.6 (1.2 – 5.4) C2. Low fruit /vegetable intake / Low physical activity / High screen time / High soft drink intake Observed % = 2.6 Expected % = 2.1 O/E (95%CI) = 1.2 (0.7 – 2.2) C3. Low fruit /vegetable intake / Low physical activity / High screen time / High snack intake Observed % = 6.6 Expected % = 4.3 O/E (95%CI) = 1.5 (1.2 – 2.0) C4. Low physical activity / High screen time / High soft drink intake / High snack intake Observed % = 3.1 Expected % = 2.2 O/E (95%CI) = 1.4 (0.9 – 2.2) C5. Low fruit /vegetable intake / Low physical activity / High screen time Observed % = 6.0 Expected % = 6.4 O/E (95%CI) = 0.9 (0.7 – 1.2) C6. Low physical activity / High screen time / High snack intake Observed % = 4.7 Expected % = 6.7 O/E (95%CI) = 0.7 (0.5 – 1.0) C7. Low physical activity / High screen time / High soft drink intake Observed % = 4.2 Expected % = 3.3 O/E (95%CI) = 1.3 (0.5 – 3.1) G ↑ co-occurrence of UHB B G with 5 UHB (C1) was 160% more than it would be expected	↑ H SES ↓ UHB ↓ L SES ↑ UHB	NA
Hartz et al., (2018) [44]	C 1B ↑MVPA ↑DQ ↓SB C 2B ↓MVPA ↓DQ ↓SB C 3B ↓MVPA ↑DQ ↑SB C 1A ↑MVPA ↑DQ ↑SB C 2A ↓MVPA ↑DQ ↓SB C 3A ↓MVPA ↓DQ ↑SB (*n* = 3)	–-	C 1B 10.3% C 2B 62% C 3B 27.8% C 1A 5.7% C 2A 49.6% C 3A 44.7%	NA	C 3B ↑ older age
Iannotti and Wang (2013) [42]	C1. Healthful ↑PA ↑FV ↓ED ↓ST C2. Unhealthful ~ PA ~ FV ↑ED ↑ST C3. Typical ↓PA ↓FV ↓ED ~ ST (*n* = 3)	C1. Healthful (26.5%) C2. Unhealthful (26.4%) C3. Typical (47.2%)	C3 ↑ B	C2 ↑ L SES C3 ↑ L SES	C3 ↑ older
Iaccarino Idelson et al., (2014) [76]	C1 ↑MVPA ↑BF ↑DP ↑FV ↓TV (*n* = 1)	C1 0% (0% of the sample met the five health recommendations, < 0.5% fulfilled four recommendations, only about 5% fulfilled three recommendations, and 21% of the sample did not meet any health recommendation.)	< 0.5% fulfilled four recommendations (0.5% boys, 0% girls) About 5% fulfilled three recommendations (3.4% boys, 5.8% girls)	NA	NA
Kerkadi et al., (2021) [82]	C1 ↓PA ↓FV ↑SB (*n* = 1)	C1 Urban Observed % = 1.8 Expected % = 1.6 O/E (95%CI) = 1.13 Rural Observed % = 1.3 Expected % = 1.6 O/E (95%CI) = 0.81	NA	NA	NA
Khan et al., (2019) [80]	C1 ↓PA ↓FV ↑SITT (*n* = 1)	–-	Boys Observed % = 7.2 Expected % = 6.7 O/E (95%CI) = 1.1 (0.8 − 1.4) Girls Observed % = 6.6 Expected % = 5.6 O/E (95%CI) = 1.2 (0.9 − 1.5)	NA	NA
Long et al., (2021) [83]	C1 ↓PA ↓FV ↑UHF ↑SB C2 ↓PA ↓FV ↑SB C3 ↓PA ↑UHF ↑SB (*n* = 3)	–-	C1 Boys (5.2%) Girls (8.2%) C2 Boys (3.6%) Girls (4.8%) C3 Boys (8.4%) Girls (15.2%)	NA	NA
Magalhães et al., (2022) [58]	Late/on time maturing adolescents C1 ↑PA ↑FV C2 ↓PA ↓FV ↓UHF ↓ST C3 ↑PA ↑UHF ↑ST Early maturing adolescents C1 ↓PA ↓UHF ↓ST C2 ↑PA ↑UHF ↑ST (*n* = 5)	–-	Late/on time maturing adolescents C1 Boys (51.8%) Girls (48.2%) C2 Boys (39.8%) Girls (60.2%) C3 Boys (42.5%) Girls (57.5%) Early maturing adolescents C1 Boys (40.4%) Girls (59.6%) C2 Boys (45.3%) Girls (54.7%)	NA	NA
Maia et al., (2018) [52]	C1 ↓PA ↓HF ↓UHF ↓SITT C2 ↑PA ↑HF ↑UHF ↑SITT (*n* = 2)	C 1 Total (57.13%) C 2 Total (42.87%)	C 1 Boys (57.78%) Girls (55.9%) C 2 Boys (42.22%) Girls (44.1%)	C2 ↑ L SES	C2 ↑ 14–15 years age group
Mandic et al., (2017) [81]	C1. Non-adherent, healthy weight ↓MVPA ↓FV ↑ST C2. Non- adherent, unhealthy weight ↓MVPA ↓FV ↑ST C3. Semi-adherent, unhealthy weight ↓PA ↑FV ↑ST C4. Physically active, healthy weight ↑PA ↓FV ↑ST C5. Low screen time, healthy weight ↓PA ~ FV ↓ST C6. Healthy F&V intake, healthy weight ↓PA ↑FV ↑ST (*n* = 6)	C1. Non-adherent, healthy weight Total (38.8%) C2. Non- adherent, unhealthy weight Total (15.4%) C3. Semi-adherent, unhealthy weight Total (11.8%) C4. Physically active, healthy weight Total (13.4%) C5. Low screen time, healthy weight Total (7.1%) C6. Healthy F&V intake, healthy weight Total (13.5%)	C1. Non-adherent, healthy weight Boys (48.9%) Girls (51.1%) C2. Non- adherent, unhealthy weight Boys (48.5%) Girls (51.5%) C3. Semi-adherent, unhealthy weight Boys (49.4%) Girls (50.6%) C4. Physically active, healthy weight Boys (65.5%) Girls (34.5%) C5. Low screen time, healthy weight Boys (32.6%) Girls (67.4%) C6. Healthy F&V intake, healthy weight Boys (41.7%) Girls (58.3%)	C1 ↑ L SES C2 ↑ L SES C4 ↓ L SES C6 ↓ L SES	C5 ↑ young individuals
Matias et al., (2018) [53]	C1. Health-promoting SB and diet ↓PA ↓UHF ↓HF ↓SB C2. Health-promoting PA and diet ↑PA ↑HF ↓UHF ↓SB C3. Health-risk ↓PA ↑UHF ↓HF ↑SB (*n* = 3)	C1. Health-promoting SB and diet (32.6%) C2. Health-promoting PA and diet (44.9%) C3. Health-risk (22.5%)	C1 ↑ G C2 ↑ B C3 ↑ G	C1 ↑ L SES C2 ↑ H SES C3 ↑ L SES C1 inversely associated SES H SES 37% less to be in C1 H SES 21% more to be in C2	↑ year in age 7% ↑ to be in C1
Moreira et al., (2018) [64]	Among boys, clusters 1 to 3, and 5, were similar in both HELENA and ELANA studies whereas cluster 4 showed different behaviours C1. Cluster ↓MVPA ↓FV ↓SSB ↑TV C2. Cluster 2 ↓MVPA ↓FV ↑SSB ~ TV C3. Cluster 3 ↑MVPA ↓FV ↓SSB ↓TV C4. Cluster 4 In the HELENA study, cluster 4 was characterized by: ↓MVPA ↑FV ↓SSB ↓TV In the ELANA study, cluster 4 was characterized by: ↑MVPA ~ FV ↑SSB ↑TV C5. Cluster 5 ↓MVPA ↓FV ↓SSB ↓TV Among girls, clusters showed similarities in both studies C1. Cluster 1 ↓MVPA ↓FV ↓SSB ↑TV C2. Cluster 2 ↓MVPA ↓FV ↑SSB ↓TV C3. Cluster 3 ↑MVPA ↓FV ↓SSB ↓TV C4. Cluster 4 ↓MVPA ↑FV ↓SSB ↓TV whereas in ELANA girls this cluster presented as: ↑MVPA ↑FV ↑SSB ↓TV C5. Cluster 5 ↓MVPA ↓FV ↓SSB ↓TV (*n* = 5)	–-	HELENA C1. Cluster 1 Boys (18.7%) Girls (21.9%) C2. Cluster 2 Boys (11.6%) Girls (13.6%) C3. Cluster 3 Boys (19.6%) Girls (15.5%) C4. Cluster 4 Boys (17.7%) Girls (18.9%) C5. Cluster 5 Boys (32.4%) Girls (30.2%) ELANA C1. Cluster 1 Boys (35.3%) Girls (34.8%) C2. Cluster 2 Boys (16.2%) Girls (11.8%) C3. Cluster 3 Boys (12.4%) Girls (12.2%) C4. Cluster 4 Boys (7.3%) Girls (5.8%) C5. Cluster 5 Boys (26.1%) Girls (35.3%)	No significant difference	HELENA B, C3 ↑ youngest age HELENA G, C3 ↑ youngest age ELANA G, C4 ↑ older age
Niermann et al., (2018) [78]	C1. Healthy behavior families ↑MVPA ↑HF ↓SW ↓ST C2. Unhealthy behavior families ↓MVPA ↓HF ↑SW ↑ST C3. Divergent behavior families ↓MVPA ↑HF ↓SW ↓ST (*n* = 3)	–-	C1. Healthy behavior families Boys (32.8%) Girls (67.2%) C2. Unhealthy behavior families Boys (50.9%) Girls (49.1%) C3. Divergent behavior families Boys (32.4%) Girls (67.6%)	C1 and C3 ↑ H SES C2 ↑ L SES	NA
Nunes et al., (2016) [54]	C1 ↓PA ↑UHF ↑ST (*n* = 1)	Total (40.5%) Observed % (95%CI) = 40.6 (37.4–43.7) Expected %(95%CI) = 38.0 (34.8–41.1) O/E (95%CI) = 1.1 (0.4–1.7)	Boys (38.4%) OR (95%CI) = 1.00 Girls (42.1%) OR (95%CI) = 1.10 (0.84–1.45)	No significant difference	C1 ↓ older individuals
Ottevaere et al., (2011) [66]	C1. Unhealthy ↓MVPA ↓DQ ↓SB C2. Sedentary ↓MVPA ~ DQ ↑SB C3. Active, low diet quality ↑MVPA ↓DQ C4. Inactive, high diet quality ↓MVPA ↑DQ ↓SB C5. Healthy ↑MVPA ↑DQ (*n* = 5)	C1. Unhealthy Total (21%) C2. Sedentary Total (12%) C3. Active, low diet quality Total (7%) C4. Inactive, high diet quality Total (42%) C5. Healthy Total (18%)	C1. Unhealthy Boys (49.5%) Girls (50.5%) C2. Sedentary Boys (51%) Girls (49%) C3. Active, low diet quality Boys (63.8%) Girls (36.2%) C4. Inactive, high diet quality Boys (35.8%) Girls (64.2%) C5. Healthy Boys (53.2%) Girls (46.8%)	L SES ↓ C4 and C5 C2 B ↑ H SES	C3 and C5 ↑ younger age C1 ↑ older B
Sanchez et al., (2007) [46]	C1. TV > 120 min per day / PA < 60 min per day / Fat > 30% fat of total energy intake / Fruits/vegetables < 5 servings/day ↓MVPA ↑F ↓FV ↑TV C2. TV > 120 min per day / PA < 60 min per day / Fat > 30% fat of total energy intake / Fruits/vegetables > 5 servings/day ↓MVPA ↑F ↑FV ↑TV C3. TV > 120 min per day / PA < 60 min per day/ Fat < 30% fat of total energy intake / Fruits/vegetables < 5 servings/day ↓MVPA ↓F ↓FV ↑TV (*n* = 3)	2% met all guidelines	C1. TV > 120 min per day / PA < 60 min per day / Fat > 30% fat of total energy intake / Fruits/vegetables < 5 servings/day Boys (9.8%) Girls (14.8%) C2. TV > 120 min per day / PA < 60 min per day / Fat > 30% fat of total energy intake / Fruits/vegetables > 5 servings/day Boys (1.1%) Girls (1.0%) C3. TV > 120 min per day / PA < 60 min per day/ Fat < 30% fat of total energy intake / Fruits/vegetables < 5 servings/day Boys (3.4%) Girls (7.8%)	NA	↑ UHB ↑ older age
Silva et al., (2014) [50]	C1 ↓MVPA ↓FV ↑ST (*n* = 1)	–	Boys Observed % = 11.8 Expected % = 9.0 O/E (95% CI) = 1.32 (1.18, 1.47) Girls Observed % = 12.2 Expected % = 11.0 O/E (95% CI) = 1.12 (1.02, 1.23)	NA	NA
Spengler et al., (2012) [77]	C1. Cluster 1 ↑PA ~ DQ ~ MU C2. Cluster 2 ↓PA ↑DQ ↓MU C3. Cluster 3 ↓PA ↓DQ ↑MU C4. Cluster 4 ↓PA ↓DQ ↓MU (*n* = 4)	C1. Cluster 1 Total (16.2%) C2. Cluster 2 Total (34.3%) C3. Cluster 3 Total (18.6%) C4. Cluster 4 Total (30.9%)	C1. Cluster 1 Boys (71.8%) Girls (28.2%) C2. Cluster 2 Boys (38.7%) Girls (61.3%) C3. Cluster 3 Boys (69.3%) Girls (30.7%) C4. Cluster 4 Boys (41.6%) Girls (58.4%)	C1 ↑ H SES C2 ↑ H SES C3 ↑ L SES	C3 ↑ older adolescents
Spengler et al., (2014) [72]	C1. Cluster 1 ↑PA ~ DQ ~ MU C2. Cluster 2 ↓PA ↑DQ ↓MU C3. Cluster 3 ↓PA ↓DQ ↑MU C4. Cluster 4 ↓PA ↓DQ ↓MU (*n* = 4)	C1. Cluster 1 (16.2%) C2. Cluster 2 (34.3%) C3. Cluster 3 (18.6%) C4. Cluster 4 (30.9%)	NA	NA	NA
Teh et al., (2019) [79]	C1 ↓PA ↓FV ↑SB (*n* = 1)	–-	Boys Observed % = 23.15 Expected % = 20.67 O/E = 1.12 Girls Observed % = 35.5 Expected % = 34.52 O/E = 1.03	NA	NA
Uddin et al., (2020) [68]	C1 ↓PA ↓FV ↑SITT (*n* = 1)	–-	Boys Observed % = 11.71 Expected % = 12.44 O/E (95%CI) = 0.94 (0.9 − 0.98) Girls Observed % = 17.73 Expected % = 16.27 O/E (95%CI) = 1.09 (1.06 − 1.12)	NA	NA
Veloso et al., (2012) [74]	C1. Active gamers ↑PA ↑SW ↑CSD ↓FV ↑ST C2. Healthy group ↑PA ↓SW ↓CSD ↑FV ↓SB C3. Sedentary group ↓PA ~ SW ~ CSD ↓FV ↓SB ~ TV (*n* = 3)	C1. Active gamers Total (25%) C2. Healthy group Total (41%) C3. Sedentary group Total (34%)	C1. Active gamers Boys (35.8%) Girls (15.3%) C2. Healthy group Boys (42.2%) Girls (40.3%) C3. Sedentary group Boys (22.1%) Girls (44.3%)	NA	C1 ↑ younger age C3 ↑ older age
Wadolowska et al., (2018) [75]	C1. Prudent-Active ↑VPA ↑HF ↑BF ↓ST C2. Fast-food-Sedentary ↑UHF ↓BF ↑ST C3. notPrudent-notFast-food-lowActive ↓VPA ↓HF ↓UHF ↓BF (*n* = 3)	C1. Prudent-Active Total (29.3%) C2. Fast-food-Sedentary Total (13.8%) C3. notPrudent-notFast-food-lowActive Total (56.9%)	C1. Prudent-Active Boys (41.9%) Girls (58.1%) C2. Fast-food-Sedentary Boys (59.3%) Girls (40.7%) C3. notPrudent-notFast-food-lowActive Boys (49.3%) Girls (50.7%)	C2 ↑ L SES	C1 ↑ younger age C2 ↑ older age
**Children and Adolescents (*n* = 7)**					
Khoshhali et al., (2021) [90]	C1 ↓FV ↓DP C2 ↑ PA ↑SW ↑SSB ↑SN ↑FF ↓FV ↓DP C3 ↑ PA ↓BF ↓FV ↓DP ↑FF ↑SW ↑SSB ↑SN (*n* = 3)	–-	C1 Boys (83.5%) Girls (81.1%) C2 Boys (4.1%) Girls (7.4%) C3 Boys (12.4%) Girls (11.5%)	No significant difference	NA
Leech et al., (2014) [60]	C1. Most healthy ↑MVPA ↓ED ↓SB ↓TV C2. ED consumers who watch TV ↑ED ↓FV ↑TV C3. High sedentary behaviour/low MVPA ↓MVPA ↑SB (*n* = 3)	Younger children C1. Most healthy Total (35%) C2. ED consumers who watch TV Total (39%) C3. High sedentary behaviour/low MVPA Total (26%) Older children C1. Most healthy Total (32%) C2. ED consumers who watch TV Total (37%) C3. High sedentary behaviour/low MVPA Total (31%)	Younger children C1. Most healthy Boys (50%) Girls (50%) C2. ED consumers who watch TV Boys (56%) Girls (44%) C3. High sedentary behaviour/low MVPA Boys (40%) Girls (59%) Older children C1. Most healthy Boys (54%) Girls (46%) C2. ED consumers who watch TV Boys (48%) Girls (52%) C3. High sedentary behaviour/low MVPA Boys (31%) Girls (69%)	C2 ↑ L SES	Younger children ↑ HB ↓ UHB in Cs C2 ↑ older children
Leech et al., (2015) [59]	C1. Most healthy ↑MVPA ↓ED ↓SB ↓TV C2. ED consumers who watch TV ↑ED ↑TV C3. High sedentary behaviour/low MVPA ↓MVPA ↑SB (*n* = 3)	5–6-year-olds, T1 C1. Most healthy (40%) C2. ED consumers who watch TV (35%) C3. High sedentary behaviour/low MVPA (25%) 5–6-year-olds, T2 C1. Most healthy (34%) C2. ED consumers who watch TV (25%) C3. High sedentary behaviour/low MVPA (41%) 10–12-year-olds, T1 C1. Most healthy (41%) C2. ED consumers who watch TV (32%) C3. High sedentary behaviour/low MVPA (26%) 10–12-year-olds, T2 C1. Most healthy (29%) C2. ED consumers who watch TV (29%) C3. High sedentary behaviour/low MVPA (42%)	C1 ↑ B C2 ↑ B C3 ↑ G C1 T1 64% younger B T2 60% older B C2 T1 61% younger B T2 68% older B C3 T1 40% younger B T2 32% older B	NA	C1 ↑ older age C2 ↑ younger age C3 ↑ younger age
Mayne et al., (2020) [19]	C1. Screen time / diet / physical activity ↓PA ↓HEI ↑ST C2. Screen time / physical activity / fast food ↓PA ↑FF ↑ST (*n* = 2)	Age 6–11 C1. Screen time / diet / physical activity (11.9%) C2. Screen time / physical activity / fast food (4.6%) Age 12–15 C1. Screen time / diet / physical activity (19.4%) C2. Screen time / physical activity / fast food (4.0%) Age 16–19 C1. Screen time / diet / physical activity (22.2%) C2. Screen time / physical activity / fast food (9.5%)	NA	NA	C1 ↑ older adolescents (22.2%)
Sánchez-Oliva et al., (2018) [71]	4 clusters (older children) C1. Healthy lifestyle ↑MVPA ~ MED ↓ST ↓SB C2. Sedentary/healthy diet ↓MVPA ↑MED ↓ST ↑SB C3. High screen ~ MVPA ↓MED ↑ST ~ SB C4. Low moderate to vigorous physical activity/unhealthy diet ↓MVPA ↓MED ↓ST ↑SB 4 clusters (younger adolescents) C1. Healthy lifestyle ↑MVPA ~ MED ↓ST ↓SB C2. Highly sedentary ~ MVPA ~ MED ~ ST ↑SB C3. High screen/ unhealthy diet ~ MVPA ↓MED ↑ST ~ SB C4. Low screen and moderate to vigorous physical activity ↓MVPA ↑MED ↓ST ~ SB 4 clusters (older adolescents) C1. Healthy lifestyle ↑MVPA ↑MED ↓ST ↓SB C2. Sedentary/ healthy diet ↓MVPA ↑MED ↓ST ↑SB C3. High screen ~ MVPA ~ MED ↑ST ~ SB C4. Low moderate to vigorous physical activity/unhealthy diet ↓MVPA ↓MED ~ ST ~ SB (*n* = 4)	Older children C1. Healthy lifestyle Total (26%) C2. Sedentary/healthy diet Total (30%) C3. High screen Total (22%) C4. Low moderate to vigorous physical activity/unhealthy diet Total (22%) Younger adolescents C1. Healthy lifestyle Total (27%) C2. Highly sedentary Total (9%) C3. High screen/ unhealthy diet Total (25%) C4. Low screen and moderate to vigorous physical activity Total (40%) Older adolescents C1. Healthy lifestyle Total (25%) C2. Sedentary/ healthy diet Total (29%) C3. High screen Total (22%) C4. Low moderate to vigorous physical activity/unhealthy diet Total (24%)	Older children C1. Healthy lifestyle Boys (38.7%) Girls (13.1%) C2. Sedentary/healthy diet Boys (22.6%) Girls (38.4%) C3. High screen Boys (16.6%) Girls (26.8%) C4. Low moderate to vigorous physical activity/unhealthy diet Boys (22.1%) Girls (21.7%) Younger adolescents C1. Healthy lifestyle Boys (41.6%) Girls (10.3%) C2. Highly sedentary Boys (7.3%) Girls (9.9%) C3. High screen/ unhealthy diet Boys (24.8%) Girls (25.9%) C4. Low screen and moderate to vigorous physical activity Boys (26.3%) Girls (53.9%) Older adolescents C1. Healthy lifestyle Boys (35%) Girls (15.8%) C2. Sedentary/ healthy diet Boys (18.6%) Girls (38%) C3. High screen Boys (28.8%) Girls (16.3%) C4. Low moderate to vigorous physical activity/unhealthy diet Boys (17.5%) Girls (29.9%)	In younger adolescents’ group, L SES ↑ C3	–-
Schmiege et al., (2016) [45]	C1. Healthiest ↑PA ↓SN ↓SSB ↑FV ↑BF ↓ST C2. Least Healthy ↓PA ↑JF ↓FV ↑ST C3. Mixed diet/low activity/low screen time ↓PA ↓JF ↓FV ↓ST C4. Mixed diet/high activity/high screen time ↑PA ↑SN ↑SSB ↑FV ↑BF ↑ST (*n* = 4)	C1. Healthiest Total (44%) C2. Least Healthy Total (7%) C3. Mixed diet/low activity/low screen time Total (37%) C4. Mixed diet/high activity/high screen time Total (11%)	C1. Healthiest Boys (55.4%) Girls (44.6%) C2. Least Healthy Boys (46.1%) Girls (53.9%) C3. Mixed diet/low activity/low screen time Boys (48.0%) Girls (52%) C4. Mixed diet/high activity/high screen time Boys (50.9%) Girls (49.1%)	NA	**Percentage of Children/Adolescents in each cluster stratified by age categories** C1. Healthiest Preschool (55.93%) School Age (45.21%) Adolescent (32.15%) C2. Least Healthy Preschool (1.36%) School Age (6.85%) Adolescent (12.54%) C3. Mixed diet/low activity/low screen time Preschool (32.88%) School Age (36.99%) Adolescent (42.12%) C4. Mixed diet/high activity/high screen time Preschool (9.83%) School Age (10.96%) Adolescent (13.18%)
Schroder et al., (2018) [89]	C1. Low physical activity / High screen time / Skipping breakfast / Low meal frequency ↓PA ↓BF ↓MF ↑ST C2. Low physical activity / High screen time / Skipping breakfast ↓PA ↓BF ↑ST C3. Low physical activity / High screen time / Low meal frequency ↓PA ↓MF ↑ST (*n* = 3)	C1. Low physical activity / High screen time / Skipping breakfast / Low meal frequency (5%) C2. Low physical activity / High screen time / Skipping breakfast (0.8%) C3. Low physical activity / High screen time / Low meal frequency (1.4%)	G ↑ UHB	L SES ↑ UHB ↓ H SES ↑ UHB	↑ UHB ↑ age
**Adolescents and young Adults (*n* = 1)**					
Watts et al., (2015) [91]	C1 ↓MPA ↓FV ↑SITT (n = 1)	C1 7.4%	NA	NA	NA
**Young Adults (*n* = 1)**					
Al-Nakeeb et al., (2015) [92]	C1. High risk factors ↓MPA ↓HF ↑UHF ↑TV C2. Moderate risk factors ↑MPA ~ HF ~ UHF ↓TVCOM C3. Low risk factors ~ MPA ↑HF ↓UHF ↑TVCOM (*n* = 3)	C1. High risk factors Total (42.2%) C2. Moderate risk factors Total (24.4%) C3. Low risk factors Total (33.3%)	C1. High risk factors Boys (29.4%) Girls (70.6%) C2. Moderate risk factors Boys (69.0%) Girls (31.0%) C3. Low risk factors Boys (41.3%) Girls (58.7%)	NA	C1 ↑ youngest age C3 ↑ oldest age

*SES* socioeconomic status, *↑* high, *↓* low, *PA* physical activity, *FV* fruits and vegetables, *DISF* discretionary foods, *ST* screen time, *B* boys, *H* high, *VPA* vigorous physical activity, *CSD* carbonated soft drink, *NA* not assessed, *MVPA* moderate to vigorous physical activity, *ED* energy dense, *MAR* mean adequacy ratio, *SSB* sugar sweetened beverages, *DQ* diet quality, *UHF* unhealthy foods, *HF* healthy foods, *–-* not available, *G* girls, *TVCOM* television and computer, *SB* sedentary behaviours, *SN* unhealthy snacks, ~ moderate, *FF* fast foods, *SBHW* sedentary behaviours devoted to homework, *FIB* fibre, *SSS* sugar, salty snacks and soda, *BF* breakfast, *DP* dairy products, *TV* television, *SITT* sitting time, *SW* sweets, *MU* media use, *HEI* healthy eating index, *MED* Mediterranean diet, *JF* junk food, *MF* meal frequency, *MPA* moderate physical activity

Twenty-two studies stratified clusters of health behaviours by sex [25, 42, 44, 46, 50–52, 55, 56, 63, 64, 66–69, 72, 76, 79, 80, 83, 89, 90], ten studies by age group [19, 45, 57, 59, 60, 63, 69, 71, 72, 89], three by region [64, 65, 82], and one study by maturity status [58]. Overall, more females were found in the unhealthy clusters [25, 44, 46, 53–55, 57, 59, 60, 68, 71, 77, 80, 83, 89, 92] or mixed clusters comprising a combination of high diet quality, low PA, and/or high SB [44, 45, 48, 51–53, 58, 63, 66, 74, 77, 81, 85, 88]. Males tended to be found in healthy [45, 58, 59, 62, 71, 77] or mixed clusters characterised by a combinations of high PA, high SB, and/or low diet quality [48, 51, 53, 56, 63, 66, 69, 70, 74, 77, 81, 86, 88, 92]. With regard to differences in age groups, most studies found that younger individuals (i.e. children, younger adolescents) tended to belong to healthier clusters [45, 51, 59, 63, 64, 75, 85], while older participants (i.e. older adolescents, young adults) were likelier to be in unhealthy [42, 45, 46, 59, 60, 63, 64, 66, 75, 88, 89] or mixed clusters characterised by lower PA [44, 46, 53, 74, 89].

Twenty-two studies assessed differences in socioeconomic status among clusters of health behaviours of interest [25, 42, 47, 48, 51–54, 57, 60, 62, 64, 66, 67, 71, 75, 77, 78, 81, 86, 89, 90]. The data suggest that young people from low socioeconomic status exhibit unhealthier lifestyle patterns compared to those from families with higher socioeconomic status [25, 51, 53, 60, 67, 70, 71, 77, 78, 81, 86, 89].

### Markers of adiposity

Sixteen studies examined associations between clusters of health behaviours and markers of adiposity (Table 2) [51, 55, 56, 59, 63–65, 67, 70–75, 89, 90]. Three examined those associations in children [65, 67, 70], nine in adolescents [51, 55, 56, 63, 64, 72–75], and four in both children and adolescents [59, 71, 89, 90]. The majority included body mass index (BMI) as the main anthropometric marker, whether continuous [59, 65, 67, 70, 73, 89] or in categories (e.g. normal weight, overweight, obese) [51, 55, 56, 59, 64, 65, 70, 72, 74, 75, 89, 90], except one study that used body fat percentage [63, 71], and one study that used body fat percentage with fat-free mass percentage, and waist circumference [63]. Some studies measured additional adiposity indicators in addition to BMI, such as waist-to-height ratio (WHtR) [75, 89], waist circumference [55, 64, 67], skinfolds [67], and body fat percentage using bio-electrical impedance [64]. Sixteen studies examined the association of behavioural clusters and adiposity; twelve cross-sectional [51, 55, 56, 63–65, 67, 73–75, 89, 90] and four longitudinal [59, 70–72]. Of the studies that examined BMI, continuous or in categories (overweight/obesity), a total of nine studies [51, 55, 64, 65, 67, 70, 74, 75, 89] found an association between clusters with mixed (low PA and/or high SB) and unhealthy behaviour clusters and increased probability of overweight/obesity, one found an unexpected inverse association [73], and two found no association [56, 59].

**Table 2:** Associations of clustering patterns of diet, PA and sedentary behaviours with adiposity in children, adolescents, and young adults (*n* = 16)

Authors (Year)	Clusters summary	Health outcomes	Method of analysis	Covariates	Sex-stratified associations	Results
**Children (*n* = 3)**						
Bel-Serrat et al., (2019) [65]	C1. Physically active and healthy diet ↑VPA ↑FV ↓CSD ↓ST C2. Healthy diet ↑FV ↓CSD *C1 and C2 were observed in all four groups C3. Physically active ↑VPA * Cluster 3 was observed in East Europe, South Europe/Mediterranean countries and West-Central Asia C4. Physically active and sedentary ↑VPA ↑ST * Cluster 4 only emerged in the North European countries C5. Sedentary and physically inactive ↓VPA ↑ST * North Europe, South Europe/Mediterranean countries and West-Central Asia C6. Low beverage intake, low sedentary and physically inactive ↓VPA ↓FV ↓CSD ↓ST *Cluster 6 was present in all the groups except in North Europe C7. High beverage intake and F&V intake ↑FV ↑CSD * Cluster 7 was present in both North Europe and West-Central Asia C8. Sedentary, physically inactive and healthy diet ↓VPA ↑FV ↓CSD ↑ST *Cluster 8 was emerged in North European countries C9. High beverage intake, sedentary and physically inactive ↓VPA ↑CSD ↑ST *Cluster 9 comprised in the North European and East European countries C10. Sedentary and physically active ↑VPA ↑ST *Cluster 10 was observed in East Europe only C11. High beverage intake, sedentary and physically active ↑VPA ↑CSD ↑ST *Cluster 11 was emerged in both East Europe and in South Europe/Mediterranean countries C12. Sedentary, physically active and healthy diet ↑VPA ↑FV ↓CSD ↑ST * Cluster 12 was specific to South Europe/Mediterranean countries C13. Physically active, high beverage intake, sedentary and high F&V intake ↑VPA ↑FV ↑CSD ↑ST *Cluster 13 was only observed in West-Central Asia	BMI/A z-scores Weight status: • Underweight/healthy weight • Overweight/obese	Mixed-effects regression	Sex, age, parental education level and season of completion of the questionnaire	No	South Europe/Mediterranean All Cs except C3 + BMI/A + overweight/obese East Europe C2, C6, C9, and C10 + BMI/A C2, C6, C9, and C10 + overweight/obese North Europe C8 + BMI/A C8 + overweight/obese C2, C4, and C5 + overweight/obese
Gubbels et al., (2012) [70]	C1. Sedentary-snacking pattern ↑UHF ↑TV C2. Healthy intake pattern ↑HF C3. Sandwich pattern ↑UHF ↑HF C4. Sporty-traditional meal pattern ↑PA ↑HF	BMI z-score Weight status: • Overweight	Backward regression	Child sex, BMI z-score at age 5 years, general appetite and activity style; parental educational level, working hours, country of birth and BMI	No	C1 + BMI at age 7 years and 8 years C1 + overweight at age 7 years
Santaliestra-Pasías et al., (2015) [67]	C1 ↑PA ↓FV ↓SSB ↓SB C 2 ↓PA ↓FV ↓SSB ↑SB C 3 ↑PA ↓FV ↓SSB ↑SB C 4 ↓PA ↓FV ↑SSB ↓SB C 5 ↓PA ↓FV ↓SSB ↓SB C 6 ↓PA ↑FV ↓SSB ↓SB	BMI z-score Waist circumference z-score Sum of skinfolds z-score	ANCOVA and logistic regression	SES and age	Yes	C2 and C3 + BMIz, WCz, and SSz B in C2 + BMIz and WCz greater than one
**Adolescents (*n* = 9)**						
Cuenca-García et al., (2013) [63]	C1. Healthy diet and active ↑MVPA ↑DQ ↓ST ~ SBHW C2. Healthy diet and academic ~ MVPA ↑DQ ~ ST ↑SBHW C3. Healthy diet and inactive ↓MVPA ~ DQ ~ ST ~ SBHW C4. Unhealthy diet and screen user ~ MVPA ↓DQ ↑ST ~ SBHW C5. Unhealthy diet and active ↑MVPA ↓DQ ~ ST ~ SBHW	Body fat percentage Fat-free mass percentage Waist circumference	ANOVA	–-	Yes	0
Cureau et al., (2018) [55]	C1 ↓MVPA ↓FIB ↑ST	Weight status: • Overweight/obesity Abdominal obesity	Poisson regression	Brazilian regions, sex, age categories, skin colour, economic index, and school type	Yes	C1 + overweight/obesity and abdominal obesity
Dantas et al., (2018) [51]	C 1 ↑PA ↓FV ↓SSB ↓SB C 2 ↓PA ↓FV ↓SSB ↑SB C 3 ↑PA ↓FV ↓SSB ↑SB C 4 ↓PA ↓FV ↑SSB ↓SB C 5 ↓PA ↓FV ↓SSB ↓SB C 6 ↓PA ↑FV ↓SSB ↓SB	Weight status: • Overweight and obesity	Binary logistic regression	Age and economy class	Yes	B in C2 63% ↑ chance to have overweight and obesity G in C2 53% ↑ chance to have overweight and obesity B in C4 51% ↑ chance to have overweight and obesity G in C4 47% ↑ chance to have overweight and obesity
de Mello et al., (2021) [56]	Whole sample C1 ↓PA ↓FV ↑SSS ↑ST C2 ↑PA ↑FV ↓SSS ↑ST Boys C1 ↓PA ↑FV ↑SSS ↑ST C2 ↑PA ↑FV ↓SSS ↑ST Girls C1 ↓PA ↑FV ↑SSS ↑ST C2 ↑PA ↓FV ↑SSS ↑ST	Weight status: • Overweight including obesity • Non-overweight including thinness and normal weight	Logistic regression	Age and maternal education	Yes	0
Moreira et al., (2018) [64]	Among boys, clusters 1 to 3, and 5, were similar in both HELENA and ELANA studies whereas cluster 4 showed different behaviours C1 ↓MVPA ↓FV ↓SSB ↑TV C 2 ↓MVPA ↓FV ↑SSB ~ TV C 3 ↑MVPA ↓FV ↓SSB ↓TV C 4 In the HELENA study, cluster 4 was characterized by: ↓MVPA ↑FV ↓SSB ↓TV In the ELANA study, cluster 4 was characterized by: ↑MVPA ~ FV ↑SSB ↑TV C 5 ↓MVPA ↓FV ↓SSB ↓TV Among girls, clusters showed similarities in both studies C 1 ↓MVPA ↓FV ↓SSB ↑TV C 2 ↓MVPA ↓FV ↑SSB ↓TV C 3 ↑MVPA ↓FV ↓SSB ↓TV C 4 ↓MVPA ↑FV ↓SSB ↓TV whereas in ELANA girls this cluster presented as: ↑MVPA ↑FV ↑SSB ↓TV C 5 ↓MVPA ↓FV ↓SSB ↓TV	BMI z-score: • Overweight (including obesity) Waist circumference z-score Body fat percentage z-score	Logistic regression	Total energy intake in both studies, SES in the HELENA study, and type of school in the ELANA study	Yes	HELENA B, C2 + WC and %BF ELANA B, C1 + WC and %BF ELANA B, C4 + WC ELANA G, C3 and C4 + BMI
Spengler et al., (2014) [72]	C 1 ↑PA ~ DQ ~ MU C 2 ↓PA ↑DQ ↓MU C 3 ↓PA ↓DQ ↑MU C 4 ↓PA ↓DQ ↓MU	Weight status: • Normal weight • Overweight (including obesity)	Multinomial logistic regression and ANOVA	–-	Yes	Weight status change C2, C3, and C4 increased overweight % from T1 to T2 C3 highest overweight % and greatest increase in T2 G in C2 and C4 increase overweight in T2 B in C3 had significant change in weight status and largest increase of overweight members Older age in C2, C3, and C4 had significant increase in overweight Greatest change in weight status over time between younger and older was in C3 Age and SES were predictors for changing in weight status C3 members were more likely to change from normal weight to overweight over a period of six years
Van der Sluis et al., (2010) [73]	C1. Healthy ↑PA ↑FV ↓SN ↓CSD ↓SB C2. Quite healthy ↑PA ~ FV ↓SN ↓CSD ~ SB C3. Quite unhealthy ~ PA ↓FV ↓SN ↓CSD ↑SB C4. Unhealthy ↓PA ↓FV ↑SN ↑CSD ↑SB	BMI (kg/m^2^)	Linear regression	Sex and parental education level	No	C4 - BMI
Veloso et al., (2012) [74]	C1. Active gamers ↑PA ↑SW ↑CSD ↓FV ↑ST C2. Healthy group ↑PA ↓SW ↓CSD ↑FV ↓SB C3. Sedentary group ↓PA ~ SW ~ CSD ↓FV ↓SB ~ TV	Weight status: • Normal weight • Overweight • Obese	ANOVA	–-	No	C2 + BMI than C1 C3 + BMI
Wadolowska et al., (2018) [75]	C1. Prudent-Active ↑VPA ↑HF ↑BF ↓ST C2. Fast-food-Sedentary ↑UHF ↓BF ↑ST C3. notPrudent-notFast-food-lowActive ↓VPA ↓HF ↓UHF ↓BF	Central obesity Weight status: • Overweight/obesity	Logistic regression	Sex, age, residence, family affluence scale, and nutrition knowledge score	No	C1 - central obesity and overweight/obesity (lowest) C2 + central obesity (highest) and overweight/obesity C3 + central obesity and overweight/obesity (highest) In C1, 47% ↓ chance of central obesity and 33% ↓ chance of overweight/obesity than in C3 In C2, 2.22% ↑ chance of central obesity than in C1
**Children and Adolescents (*n* = 4)**						
Khoshhali et al., (2021) [90]	C1 ↓FV ↓DP C2 ↑ PA ↑SW ↑SSB ↑SN ↑FF ↓FV ↓DP C3 ↑ PA ↓BF ↓FV ↓DP ↑FF ↑SW ↑SSB ↑SN	Actual and perceived weight status	Multilevel logistic regression	Age, living area (urban vs. rural), child physical activity, number of children in the home, screen time, mother education, father education, parent physical activity, and parent weight status	Yes	Compared to C1, OR of having C2 for G who perceived themselves as overweight/obese were less than those who perceived themselves as normal weight, and G who their parent perceives them as overweight/obese was more than those who their parent perceives them as normal Underweight G were 37% more likely to be in C3 rather than normal-weight G Compared to C1, OR of having C2 for B who their parent perceives them as underweight was more than those who their parent perceives them as normal B who their parent perceives them as overweight/obese were 27% more likely to be in C3 rather than those who their parents perceive them as normal Significantly ↓ scores of UHB for G and B who perceived themselves as overweight/obese
Leech et al., (2015) [59]	C1. Most healthy ↑MVPA ↓ED ↓SB ↓TV C2. ED consumers who watch TV ↑ED ↑TV C3. High sedentary behaviour/low MVPA ↓MVPA ↑SB	BMI z-score Weight status: • Healthy weight • Overweight/obese	Cross-sectional and longitudinal linear and logistic regression	Sex, age group, maternal education, and clustering by school Longitudinal models were additionally adjusted for baseline BMI Z-score and baseline weight status, respectively	No	Baseline cluster 0 BMI z-score or weight status C2 at baseline ↑odds of overweight/obese at follow-up
Sánchez-Oliva et al., (2018) [71]	4 clusters (older children) C1. Healthy lifestyle ↑MVPA ~ MED ↓ST ↓SB C2. Sedentary/healthy diet ↓MVPA ↑MED ↓ST ↑SB C3. High screen ~ MVPA ↓MED ↑ST ~ SB C4. Low moderate to vigorous physical activity/unhealthy diet ↓MVPA ↓MED ↓ST ↑SB 4 clusters (younger adolescents) C1. Healthy lifestyle ↑MVPA ~ MED ↓ST ↓SB C2. Highly sedentary ~ MVPA ~ MED ~ ST ↑SB C3. High screen/ unhealthy diet ~ MVPA ↓MED ↑ST ~ SB C4. Low screen and moderate to vigorous physical activity ↓MVPA ↑MED ↓ST ~ SB 4 clusters (older adolescents) C1. Healthy lifestyle ↑MVPA ↑MED ↓ST ↓SB C2. Sedentary/ healthy diet ↓MVPA ↑MED ↓ST ↑SB C3. High screen ~ MVPA ~ MED ↑ST ~ SB C4. Low moderate to vigorous physical activity/unhealthy diet ↓MVPA ↓MED ~ ST ~ SB	Body fat percentage	Linear regression	Maternal education, accelerometer wear time, and sex	No	Older children in C1 ↓ BF% at baseline and 2 years later Younger adolescents in C1 ↓ BF% at baseline and 2 years later compared to C3 and C4 Younger adolescents in C1 greater ↓ BF% 2 years later compared to C3 and C4 (BF% changes) Older adolescents in C1 ↓ BF% at baseline compared to C2 BF% at baseline positively predicted BF% 2 years follow-up
Schroder et al., (2018) [89]	C1. Low physical activity / High screen time / Skipping breakfast / Low meal frequency ↓PA ↓BF ↓MF ↑ST C2. Low physical activity / High screen time / Skipping breakfast ↓PA ↓BF ↑ST C3. Low physical activity / High screen time / Low meal frequency ↓PA ↓MF ↑ST	BMI z-score WHtR Weight status: • Overweight • Obesity Abdominal obesity	General linear models and logistic regression	General linear models adjusted for sex, age, region, community size, maternal education, energy, and energy over and underreporting (BMI z-score and WHtR) Logistic regression adjusted for sex, age, region, community size, maternal education, energy, and energy over and underreporting (overweight and obesity and abdominal obesity)	Yes	A difference of 0.50 SD for BMI z-score and of 0.025 for WHtR between participants with no UHB and those with three or more ↑ UHB ↑ odds of overweight and abdominal obesity 3 UHB ↑ odds of overweight by 168% and ↑ odds of abdominal obesity by 112%, compared to those with none

*↑* high, *VPA* vigorous physical activity, *FV* fruits and vegetables, *↓* low, *CSD* carbonated soft drink, *ST* screen time, *BMI/A* body mass index for age, + positive significant associations reported, *UHF* unhealthy foods, *TV* television, *HF* healthy foods, *PA* physical activity, *BMI* body mass index, *SSB* sugar sweetened beverages, *SB* sedentary behaviours, *SES* socioeconomic status, *BMIz* body mass index z-score, *WCz* Waist circumference z-score, *SSz* Sum of skinfolds z-score, *B* boys, *MVPA* moderate to vigorous physical activity, *DQ* diet quality, ~ moderate, *SBHW* sedentary behaviours devoted to homework, *0* No significant associations reported, *FIB* fibre, *G* girls, *SSS* sugar, salty snacks and soda, *WC* Waist circumference, *%BF* percent body fat, *MU* media use, *SN* unhealthy snacks,—negative/inverse significant associations reported, *SW* sweets, *BF* breakfast, *DP* dairy products, *FF* fast foods, *OR* odds ratio, *ED* energy dense, *MED* Mediterranean diet, *MF* meal frequency

Three longitudinal studies [59, 70, 72] examined associations between clusters and BMI and concluded that high TV viewing, high energy-dense food/drink consumption [59] and high SB and unhealthy snacks [70], resulted in a higher likelihood of being classified as overweight/obese [59] and increased BMI [70]. One study found that unhealthy clusters characterised by high media use (i.e., watching TV, using a computer, and playing console games) and low PA and diet quality resulted in the greatest difference in weight status and was related to change from normal weight to overweight over time [72].

One study investigating the clustering of health behaviours across groups of countries (North and East Europe, South Europe/Mediterranean Countries, and West-Central Asia) found that unhealthy and mixed clusters were positively associated with a greater risk of being overweight or obese compared to healthy clusters [65]. Another study conducted in eight European countries (Italy, Estonia, Cyprus, Belgium, Sweden, Hungary, Germany and Spain) found that increased time in sedentary activities and low PA was associated with higher BMI, but only in boys [67].

All studies that examined mixed and unhealthy clusters showed positive associations with waist circumference [55, 64, 67, 75, 89]. Although the findings of these studies varied; for example, one found an association only among boys [67] and another found that the risk of central obesity was over double among those in an unhealthy cluster (high fast foods, sweetened beverages, energy drinks and sweets, and breakfast or school meal skipped and had screen time more 10 h/day) compared to those in a healthy cluster (high FV, dairy products and fish, consumed daily breakfast or school meal, had VPA and low screen time) [75]. Another study showed that young people in the healthy cluster (low screen time and SB, high MVPA, and average to high levels of adherence to Mediterranean diet) had considerably lower body fat at baseline and two years later, with body fat percentage at baseline being a positive predictor of body fact percentage two years later for all groups [71]. However, no significant differences by cluster were noted in body composition [63] or skin folds [67].

### Cardiometabolic outcomes

Five studies examined associations between clusters of health behaviours and cardiometabolic markers, including aerobic fitness (Table 3) [43, 44, 58, 63, 69]. One study found that girls and boys in the healthy cluster (high diet quality and MVPA and low screen use) had higher aerobic fitness levels [63], whereas boys in the unhealthy cluster —high screen time and low diet quality—had the lowest aerobic capacity compared to other clusters [63]. In another study, there were no associations between clusters and cardiorespiratory fitness (VO_2_ max) in girls, but boys in the healthy cluster (high diet score and MVPA and screen time) had the highest VO_2_ max [44]. Furthermore, another study found that clusters with low SSB consumption and/or low levels of screen time were associated with a healthier cardiovascular disease (CVD) profile than being physically active or eating a diet high in FV [69]. However, clusters of self-reported diet, screen-time and PA were not related to CVD risk in children in one study [43]. One study examined the association with metabolic risk in adolescents according to maturity status and found that early-maturing adolescents in the cluster with a greater number of healthy behaviours but less PA had a greater metabolic risk score [58].

**Table 3:** Associations of clustering patterns of diet, PA and sedentary behaviours with CVD and health-related fitness in children, adolescents, and young adults (*n* = 5)

Authors (Year)	Clusters summary	Health outcomes	Method of analysis	Covariates	Sex-stratified associations	Results
**Children (*n* = 2)**						
Bel-Serrat et al., (2013) [69]	C1. Physically active ↑PA ↓FV ↓SSB ↓TV C2. Sedentary ↓PA ↓FV ↓SSB ↑TV C3. Physically active and sedentary (boys) ↑PA ↓FV ↓SSB ↑TV C3. High beverage consumption (girls) ↓PA ↓FV ↑SSB ↓TV C4. Healthy diet ↓PA ↑FV ↓SSB ↓TV C5. Low beverage consumption and low sedentary ↓PA ↓FV ↓SSB ↓TV	Individual CVD risk factors (sum two skinfolds, SBP, HOMA index, ratio TC/HDL-C, and TG) and age- and sex-specific CVD risk score	Multiple linear regression	Parental socio-economic status and study centre	Yes	G in C2 and B in C3 + HOMA B in C2 + sum of two skinfolds B in C4 + ratio TC/HDL-c B in C1 to C4 + SBP B in Cs 2,3, and 4 + CVD risk
Drenowatz et al., (2012) [43]	C1. Low PA/high ST/low diet ↓MVPA ↓DQ ↑ST C2. Low PA/high ST/high diet ↓MVPA ↑DQ ↑ST C3. High PA/low ST/low diet ↑MVPA ↓DQ ↓ST C4. High PA/low ST/high diet ↑MVPA ↑DQ ↓ST	CVD risk score	ANCOVA	Sex and total caloric intake	No	0
**Adolescents (*n* = 3)**						
Cuenca-García et al., (2013) [63]	C1. Healthy diet and active ↑MVPA ↑DQ ↓ST ~ SBHW C2. Healthy diet and academic ~ MVPA ↑DQ ~ ST ↑SBHW C3. Healthy diet and inactive ↓MVPA ~ DQ ~ ST ~ SBHW C4. Unhealthy diet and screen user ~ MVPA ↓DQ ↑ST ~ SBHW C5. Unhealthy diet and active ↑MVPA ↓DQ ~ ST ~ SBHW	Health-related fitness (20-m shuttle run z-score, handgrip strength z-score, standing broad jump z-score, and shuttle run 4 × 10 m z-score)	ANOVA	–-	Yes	B in C1 + aerobic capacity and speed-agility G in C1 + aerobic capacity, muscular strength, and speed-agility B in C1 + aerobic capacity than C2, C3, C4, and C5 and + speed-agility than B in C4 B in C4 - aerobic capacity than other Cs (worst) G in C1 + aerobic capacity, muscular strength, and speed-agility than C2, C3, and C4 G in C4 - lower-body muscular strength and speed-agility than C5 (worst) Active adolescents in C1 and C5 had statistically significant differences on aerobic capacity despite an equal z-score on MVPA (higher) and an unequal z-score on DQ (high in C1 and low in C5)
Hartz et al., (2018) [44]	C 1B ↑MVPA ↑DQ ↓SB C 2B ↓MVPA ↓DQ ↓SB C 3B ↓MVPA ↑DQ ↑SB C 1A ↑MVPA ↑DQ ↑SB C 2A ↓MVPA ↑DQ ↓SB C 3A ↓MVPA ↓DQ ↑SB	Cardiorespiratory fitness	Multivariate linear regression	Accelerometer wear time, BMI, age, race, and PIR	Yes	C 3B - VO^2^ max
Magalhães et al., (2022) [58]	Late/on time maturing adolescents C1 ↑PA ↑FV C2 ↓PA ↓FV ↓UHF ↓ST C3 ↑PA ↑UHF ↑ST Early maturing adolescents C1 ↓PA ↓UHF ↓ST C2 ↑PA ↑UHF ↑ST	Metabolic risk score	Crude linear regression	Age and chronological age	No	C1 in early maturing adolescents + metabolic risk score in comparison with C2

*↑* high, *PA* physical activity, *↓* low, *FV* fruits and vegetables, *SSB* sugar sweetened beverages, *TV* television, *CVD* cardiovascular disease, *SBP* systolic blood pressure, *HOMA* homoeostatic assessment model, *TC* total cholesterol, *HDL-C* high-density lipoprotein cholesterol, *TG* triglycerides, *G* girls, *B* boys, + positive significant associations reported, *MVPA* moderate to vigorous physical activity, *DQ* diet quality, *ST* screen time, *0* No significant associations reported, ~ moderate, *SBHW* sedentary behaviours devoted to homework,—negative/inverse significant associations reported, *SB* sedentary behaviours, *BMI* body mass index, *PIR* poverty-to-income ratio, *VO*^*2*^* max* maximum rate of oxygen consumption, *UHF* unhealthy foods

### Mental health outcomes

Mental health outcomes were examined in one study which showed that children in the healthy cluster had better self-regulation, motivation, communication with parents, and liking school compared to those in a mixed clusters [74]. Furthermore, those in a mixed cluster characterised by high PA and high SB and poor diet had better relationships with classmates than their peers in a mixed cluster characterised by low PA and low FV and moderate TV.

## Discussion

The aim of this systematic review was to synthesize evidence on the prevalence of clusters of PA, SB, and dietary behaviours and to examine their associations with physical and mental health outcomes in children, adolescents, and young adults aged 5–24 years. Health behaviour patterns by age, sex, and socioeconomic status were also examined. The health behaviour clusters in this review were classified as healthy, unhealthy or mixed (the co-occurrence of both healthy and unhealthy behaviours). Overall, the majority of participants examined fell into the mixed clusters, which is in line with previous reviews’ findings [38, 93, 94], and supports the need for multicomponent interventions addressing several unhealthy behaviours simultaneously. It is also noteworthy that high PA and high SB most frequently clustered together, refuting the displacement hypothesis that assumes that time spent on one activity cannot be spent on another (i.e., SB displaces PA) [95]. Supportive of our findings, a previous review examined the association between SB and PA in young people and concluded that these behaviours do not directly displace one another and should be seen as different constructs [96].

Almost a quarter of clusters identified in the present review were classified as ‘unhealthy’. Clusters characterised by high SB and low PA were commonly reported across the studies. Previous reviews have shown similarly high numbers of unhealthy clusters. For example, Leech et al. found that eight of eighteen studies identified unhealthy clusters [93]. The present review also showed the coexistence of unhealthy food intake (e.g., snacks, sweets, soft drinks, junk food, SSB) with high SB (i.e., TV, media use, and/or computer use). These clusters were found in a previous review that found that SB, particularly high TV viewing, was associated with greater intake of discretionary foods and less FV consumption in children age 5–11 years [97]. The mechanisms of the observed associations between SB and diet have been examined previously and may be explained by the stimulating influence of commercials/advertisements for intake of foods high in fat, sugar and salt (HFSS) [98], that sedentary activities encourage passive snacking or overeating [99], and that watching TV while eating may disrupt habituation to food cues [100]. Not surprisingly, children were more likely to be overweight or obese in clusters with both high levels of SB and high consumption of unhealthy foods or poor diet quality. These results are in agreement with a review conducted by Leech et al. (2014), who showed that TV viewing in combination with energy-dense food and drink consumption were associated with overweight and obesity among Australian children [93]. Based on these findings, prevention programs should identify strategies aimed at uncoupling the combination of unhealthy dietary habits in front of screens.

Healthy clusters characterised by high MVPA, low screen time and overall high diet quality (e.g., high FV, low SSB, etc.), followed by high PA, low screen time and overall high diet quality were most prevalent. These findings are similar to the healthy clusters identified in Leech et al.’s (2014) review that observed these clusters in children and adolescents [93]. Another important finding in the present review that extends the findings of previous reviews was that children and adolescents with these healthy clusters had lower BMI and higher fitness compared to those in mixed and unhealthy clusters. On the other hand, more active adolescents with unfavourable diet quality showed lower fitness than those with similar activity levels but favourable diet quality [63]. It is important to note that PA might be protective of increases in adiposity and might increase fitness level when combined with a healthy diet and/or low screen time. This finding is informative for future interventions that should combine strategies for increasing PA and healthy diets while simultaneously including strategies to lower screen time. In addition, a notable finding was that only one study examined mental health outcomes of clusters. This study found that healthy clusters (high PA and FV, low sweets and soft drinks, and lowest SB) were associated with better mental health outcomes compared to mixed and unhealthy clusters. It is worth noting that in this one study PA appeared to be associated with better mental health outcomes on its own, as clusters with high PA and high SB were more strongly associated with mental health outcomes than clusters with low PA and high SB. Further research is needed to examine a range of mental health outcomes of clusters of lifestyle behaviours.

This review found that both unhealthy clusters and mixed clusters that include either high SB, low PA, and/or high SSB, alongside healthy behaviours, were associated with higher adiposity in young people. This was contrary to findings from a previous systematic review [38], which concluded that only unhealthy clusters were associated with higher adiposity levels. It is worth highlighting that in the present review only a particular combination of health behaviours within mixed clusters (i.e., those that include either high SB, low PA and/or high SSB alongside healthy behaviours) were associated with unfavourable weight status. Previous research has suggested that the combination of healthy and unhealthy behaviours negate each other’s health effects [94]. These findings suggest that, to decrease obesity risk, future research should focus on understanding in who and why, where and when such behaviours cluster together with a view to informing future multicomponent/multi-behaviour interventions to uncouple unhealthy behaviours.

In this review, clusters characterised by high SB, particularly screen time, were found to increase the risk of adiposity, irrespective of being combined with other healthy or unhealthy behaviours. As a result, it is likely that excessive screen time may reduce the beneficial effects of PA and a healthy diet on the risk of obesity. These results match those cited in Leech et al.’s (2014) review, which found a positive association between overweight and high SB [93]. The current review also found that clusters with higher screen time had greater risk of individual and clustered cardiovascular risk scores, which were predominantly seen in older boys, worse fitness levels, and greater psychosocial risks, mostly in girls. In line with this, a recent systematic review that examined the relationship between SB and health indicators in young people aged 5–17 years found that an increase in SB, expressed as total hours of screen time, was associated with an increase in cardiovascular risk markers in children and adolescents [101]. Despite PA having previously being linked with clustered metabolic risk in children [102], this review suggested that SB, expressed as screen time, has a greater role in the cardiovascular risk profile than PA. This is alarming, given the growing body of evidence to suggest that SB is independently and positively related to poor health outcomes [103]. Future public health plans need to target a reduction in screen time among young people and could be considered within multicomponent/complex interventions.

Young people’s gender, age, and socioeconomic status have consistently been shown to be associated with health behaviour cluster scores, underlining the need for tailoring prevention and intervention efforts for groups at risk. In the current review, girls tended to be in unhealthy clusters or mixed clusters mostly defined by better diet quality, but lower PA compared to boys. However, boys were almost equally distributed between unhealthy and healthy clusters or mixed clusters with high PA, unhealthy diets and/or high SB. These results can be explained by sex differences in dietary habits as hypothesized in a previous study [104], in addition to unbalanced patterns of PA [105]. Moreover, younger people (for example, children and young adolescents) and those from higher socioeconomic status were found in healthy clusters or mixed clusters with higher PA, in contrast with older people (for example, older adolescents and young adults) and those from lower socioeconomic status who were found in unhealthy clusters or mixed clusters with lower PA. These findings were consistent with previous reviews [93, 94]. A longitudinal study concluded that children’s behaviours tended to shift to unhealthier clusters with aging [59], which is in line with other longitudinal studies that show an age-related increase in SB and decrease in PA [106]. At present many monitoring efforts, such as the National Child Measurement Programme [107] in the UK, policy and interventions target children and young adolescents [108]. Given the results of this intervention there is a need to also focus efforts on older adolescents. Furthermore, the findings that socioeconomic status were negatively associated with adiposity and positively associated with health, provides further evidence for the need to devote more resources to policies and programmes targeting lower socioeconomic families.

## Strengths and limitations

To the best of our knowledge, this was the first study to systematically review clusters of PA, SB, and diet in children, adolescents, and young adults and the associations with physical and mental health outcomes. This information will be valuable for designing intervention strategies to improve the health of young people. Limitations that must be considered when interpreting these results include that a meta-analysis was not possible due to heterogeneity in the measures and analyses used in the studies included. The majority of studies were of a cross-sectional nature, which did not allow us to draw causal relationships. Also, data on many behaviours were obtained via self-report tools (such as questionnaires), which are subject to measurement errors due to social desirability or recall bias [109].

## Conclusion

This review synthesised the evidence on the prevalence of clustering of PA, SB, and diet in 5- to 24-year-olds and examined physical and mental health outcomes associated with the clusters. Clusters of health behaviours appeared to differ across socio-demographic groups and were broadly grouped into healthy, unhealthy, and mixed lifestyle clusters, emphasising the complexity and diversity across the populations examined. Mixed clusters were the most prevalent, and both mixed and unhealthy clusters were related to poor health in young people. The complex nature of these findings’ stresses the need for more research examining, in more detail, the sociodemographic factors that influence different clusters of behaviours and how these influence health. More studies that include young adults are needed.

### Supplementary Information

**Additional file 1.** 

**Additional file 2.** 

**Additional file 3.**

## Data Availability

The datasets used and/or analysed during the current study are available from the corresponding author on reasonable request.

## Abbreviations

SB: Sedentary behaviour
PA: Physical activity
FV: Fruits and vegetables
MVPA: Moderate to vigorous physical activity
PRISMA: Preferred reporting items for systematic reviews and meta-analysis
PROSPERO: International prospective register of systematic reviews
PECO: Population exposure context outcome
MPA: Moderate physical activity
VPA: Vigorous physical activity
SSB: Sugar-sweetened beverages
O/E: Observed-over-expected ratio
BMI: Body mass index
WHtR: Waist-to-height ratio
CVD: Cardiovascular disease
HFSS: High in fat, sugar and salt
