# Supplementary material for: Clustering of diet, physical activity and sedentary behaviour and related physical and mental health outcomes: a systematic review
Source: BMC Public Health. 2023 Aug 18;23:1572. doi: 10.1186/s12889-023-16372-6 (PMC10436445; doi:10.1186/s12889-023-16372-6)
Supplement: Supplementary file 1 — Additional file 1. [file 12889_2023_16372_MOESM1_ESM.docx]

**Database Search Strategy (Medline, Web of Science, and Scopus)**

**Search Strategy for MEDLINE**

1. (((((((((children [mh]) OR (child [mh])) OR (child*[Title/Abstract])) OR (kids[Title/Abstract])) OR (childhood[Title/Abstract])) OR (boy*[Title/Abstract])) OR (paediatric[Title/Abstract])) OR (girl*[Title/Abstract])) OR (early years[Title/Abstract])) OR (School age[Title/Abstract])
2. (((((((adolescents [mh]) OR (Adolescen*[Title/Abstract])) OR (young person[Title/Abstract])) OR (Teenager*[Title/Abstract])) OR (young people[Title/Abstract])) OR (Youth*[Title/Abstract])) OR (Teen*[Title/Abstract])) OR (young populations[Title/Abstract])
3. (((((young adults[Title/Abstract]) OR (adults [mh])) OR (male[Title/Abstract])) OR (female[Title/Abstract])) OR (men[Title/Abstract])) OR (women[Title/Abstract])
4. #1 OR #2 OR #3
5. ((((((((((((((clustering [mh]) OR (clustering patterns[Title/Abstract])) OR (clusters[Title/Abstract])) OR (patterns[Title/Abstract])) OR (behavioural patterns[Title/Abstract])) OR (behaviour patterns[Title/Abstract])) OR (lifestyle patterns[Title/Abstract])) OR (Behaviour [mh])) OR (cluster analysis[Title/Abstract])) OR (health behaviour[Title/Abstract])) OR (habits[Title/Abstract])) OR (Clustering[Title/Abstract])) OR (Co-existing[Title/Abstract])) OR (co-occuring[Title/Abstract])) OR (synergistic[Title/Abstract])
6. ((((((((((((((((((((((dietary behaviours[Title/Abstract]) OR (diet [mh])) OR (Soft drink[Title/Abstract])) OR (nutrition[Title/Abstract])) OR (snacks[Title/Abstract])) OR (snack*[Title/Abstract])) OR (unhealthy foods[Title/Abstract])) OR (foods[Title/Abstract])) OR (eating[Title/Abstract])) OR (snacking[Title/Abstract])) OR (energy intake[Title/Abstract])) OR (sweet drinks[Title/Abstract])) OR (fruits[Title/Abstract])) OR (unhealthy snacks[Title/Abstract])) OR (vegetables[Title/Abstract])) OR (sugar sweetened beverag*[Title/Abstract])) OR (fruit*[Title/Abstract])) OR (Energy-dense snack*[Title/Abstract])) OR (vegetable*[Title/Abstract])) OR (sweet snacks[Title/Abstract])) OR (savoury snacks[Title/Abstract])) OR (junk food[Title/Abstract])) OR (energy-dense[Title/Abstract])
7. ((((((((((((((((((((((((((((((((((((((((((physical activity [mh]) OR (exercise[Title/Abstract])) OR (physical*[Title/Abstract])) OR (active*[Title/Abstract])) OR (physical fitness[Title/Abstract])) OR (motor activity[Title/Abstract])) OR (motor active[Title/Abstract])) OR (Activit*[Title/Abstract])) OR (physically active[Title/Abstract])) OR (movement[Title/Abstract])) OR (physical exercise[Title/Abstract])) OR (movement behaviours[Title/Abstract])) OR (accelerometer[Title/Abstract])) OR (physical effort[Title/Abstract])) OR (physical exertion[Title/Abstract])) OR (Exercise Movement Techniques[Title/Abstract])) OR (Exercise Therapy[Title/Abstract])) OR (Physical Education[Title/Abstract] AND Training[Title/Abstract])) OR (aerobic train*[Title/Abstract])) OR (aerobic active*[Title/Abstract])) OR (sport*[Title/Abstract])) OR (bicycle*[Title/Abstract])) OR (swim*[Title/Abstract])) OR (walk*[Title/Abstract])) OR (run*[Title/Abstract])) OR (jog*[Title/Abstract])) OR (Physical* adj2 activ*[Title/Abstract])) OR (play[Title/Abstract])) OR (playthings[Title/Abstract])) OR (Outdoor*[Title/Abstract])) OR (activ*[Title/Abstract])) OR (activ* adj3 play*[Title/Abstract])) OR (outdoor* adj3 play*[Title/Abstract])) OR (playground*[Title/Abstract])) OR (active adj3 behaviour*[Title/Abstract])) OR (active adj3 transport*[Title/Abstract])) OR (active adj3 space*[Title/Abstract])) OR (active adj3 commut*[Title/Abstract])) OR (active adj3 neighbourhood*[Title/Abstract])) OR (active adj3 park*[Title/Abstract])) OR (active adj3 game*[Title/Abstract])) OR (active adj3 gaming[Title/Abstract])) OR (active adj3 lifestyle[Title/Abstract])
8. (((((((((((((((((((((((((((((((((((((((((((((((sedentary behaviour [mh]) OR (sedentariness[Title/Abstract])) OR (Sedentary[Title/Abstract])) OR (sedentary lifestyle[Title/Abstract])) OR (tv[Title/Abstract])) OR (television viewing[Title/Abstract])) OR (inactiv*[Title/Abstract])) OR (sitting[Title/Abstract])) OR (screen time[Title/Abstract])) OR (screentime[Title/Abstract])) OR (recreational screen time[Title/Abstract])) OR (TV viewing[Title/Abstract])) OR (lack adj2 activity[Title/Abstract])) OR (low adj3 energy expend*[Title/Abstract])) OR (computer adj time[Title/Abstract])) OR (Chair adj time[Title/Abstract])) OR (Stroller adj time[Title/Abstract])) OR (Car adj time[Title/Abstract])) OR (automobile* adj time[Title/Abstract])) OR (Auto adj time[Title/Abstract])) OR (Bus adj time[Title/Abstract])) OR (motor vehicle* adj time[Title/Abstract])) OR (indoor* adj time[Title/Abstract])) OR (in-door* adj time[Title/Abstract])) OR (Screen adj time[Title/Abstract])) OR (Television[Title/Abstract])) OR (Computers[Title/Abstract])) OR (computer gam*[Title/Abstract])) OR (electronic gam*[Title/Abstract])) OR (Video gam*[Title/Abstract])) OR (videogam*[Title/Abstract])) OR (Software[Title/Abstract])) OR (Videodisc Recording[Title/Abstract])) OR (Internet[Title/Abstract])) OR (Communications Media[Title/Abstract])) OR (handheld[Title/Abstract])) OR (Mass Media[Title/Abstract])) OR (watch* adj2 dvd*[Title/Abstract] OR video*[Title/Abstract])) OR (view* adj2 dvd*[Title/Abstract] OR video*[Title/Abstract])) OR (screen media[Title/Abstract])) OR (social media[Title/Abstract])) OR (screen based entertainment[Title/Abstract])) OR (screen-based entertainment[Title/Abstract])) OR (smartphone*[Title/Abstract])) OR (ipad[Title/Abstract])) OR (apps[Title/Abstract])) OR (app[Title/Abstract])) OR (mobile applications[Title/Abstract])
9. #6 AND #7 AND #8
10. #4 AND #5 AND #9

**Search Strategy for Web of Science**

1. TS= children OR TS= child OR TS= child* OR TS= kids OR TS= childhood OR TS= boy* OR TS= paediatric OR TS= girl* OR TS= early years OR TS= School age
2. TS= adolescents OR TS= Adolescen* OR TS= young person OR TS= Teenager* OR TS= young people OR TS= Youth* OR TS= Teen* OR TS= young populations
3. TS= young adults OR TS= adults OR TS= male OR TS= female OR TS= men OR TS= women
4. #1 OR #2 OR #3
5. TS= clustering OR TS= clustering patterns OR TS= clusters OR TS= patterns OR TS= behavioural patterns OR TS= behaviour patterns OR TS= lifestyle patterns OR TS= Behaviour OR TS= cluster analysis OR TS= health behaviour OR TS= habits OR TS= Co-existing OR TS= co-occuring OR TS= synergistic
6. TS= dietary behaviours OR TS= diet OR TS= Soft drink OR TS= nutrition OR TS= snacks OR TS= snack* OR TS= unhealthy foods OR TS= foods OR TS= eating OR TS= snacking OR TS= energy intake OR TS= sweet drinks OR TS= fruits OR TS= unhealthy snacks OR TS= vegetables OR TS= sugar sweetened beverag* OR TS= fruit* OR TS= Energy-dense snack* OR TS= vegetable* OR TS= sweet snacks OR TS= savoury snacks OR TS= junk food OR TS= energy-dense
7. TS= physical activity OR TS= exercise OR TS= physical* OR TS= active* OR TS= physical fitness OR TS= motor activity OR TS= motor active OR TS= Activit* OR TS= physically active OR TS= movement OR TS= physical exercise OR TS= movement behaviours OR TS= accelerometer OR TS= physical effort OR TS= physical exertion OR TS= “Exercise Movement Techniques“ OR TS= “Exercise Therapy” OR TS= “Physical Education and Training” OR TS= aerobic train* OR TS= aerobic active* OR TS= sport* OR TS= bicycle* OR TS= swim* OR TS= walk* OR TS= run* OR TS= jog* OR TS= (Physical* NEAR/2 "activ*") OR TS= play OR TS= playthings OR TS= Outdoor* OR TS= activ* OR TS= (activ* NEAR/3 "play*") OR TS= (outdoor* NEAR/3 "play*") OR TS= playground* OR TS= (active NEAR/3 "behaviour*") OR TS= (active NEAR/3 "transport*") OR TS= (active NEAR/3 "space*") OR TS= (active NEAR/3 "commut*") OR TS= (active NEAR/3 "neighbourhood*") OR TS= (active NEAR/3 "park*") OR TS= (active NEAR/3 "game*") OR TS= (active NEAR/3 "gaming") OR TS= (active NEAR/3 "lifestyle")
8. TS= sedentary behaviour OR TS= sedentariness OR TS= Sedentary OR TS= sedentary lifestyle OR TS= tv OR TS= television viewing OR TS= inactiv* OR TS= sitting OR TS= screen time OR TS= screentime OR TS= recreational screen time OR TS= TV viewing OR TS= (lack NEAR/2 "activity") OR TS= (low NEAR/3 "energy expend*") OR TS= (computer NEAR "time") OR TS= (Chair NEAR "time") OR TS= (Stroller NEAR "time") OR TS= (Car NEAR "time") OR TS= (automobile* NEAR "time") OR TS= (Auto NEAR "time") OR TS= (Bus NEAR "time") OR TS= (motor vehicle* NEAR "time") OR TS= (indoor* NEAR "time") OR TS= (in-door* NEAR "time") OR TS= (Screen NEAR "time") OR TS= Television OR TS= Computers OR TS= computer gam* OR TS= electronic gam OR TS= Video gam* OR TS= videogam* OR TS= Software OR TS= Videodisc Recording OR TS= Internet OR TS= Communications Media OR TS= handheld OR TS= Mass Media OR TS= (watch* NEAR/2 "dvd* or video*") OR TS= (view* NEAR/2 "dvd* or video*") OR TS= screen media OR TS= social media OR TS= screen based entertainment OR TS= "screen-based entertainment" OR TS= smartphone* OR TS= iPad OR TS= apps OR TS= app OR TS= mobile applications
9. #6 AND #7 AND #8
10. #4 AND #5 AND #9

**Search Strategy for Scopus**

| 1. TITLE-ABS-KEY ( children  OR  child  OR  child*  OR  kids  OR  childhood  OR  boy*  OR  paediatric  OR  girl* ) |
| --- |
| 1. TITLE-ABS-KEY ( early  AND  years ) |
| 1. TITLE-ABS-KEY ( school AND age ) |
| 1. #1  AND  #2  AND  3# |
| 1. TITLE-ABS-KEY ( adolescents  OR  adolescen*  OR  teenager*  OR  youth*  OR  teen* ) |
| 1. TITLE-ABS-KEY ( young  AND  person ) |
| 1. TITLE-ABS-KEY ( young  AND  people ) |
| 1. TITLE-ABS-KEY ( young  AND  populations ) |
| 1. #5  AND  #6  AND  #7  AND  #8 |
| 1. TITLE-ABS-KEY ( young  AND  adults ) |
| 1. TITLE-ABS-KEY ( adults  OR  male  OR  female  OR  men  OR  women ) |
| 1. #10  AND  #11 |
| 1. #4  OR  #9  OR  #12 |
| 1. TITLE-ABS-KEY ( clustering  AND  patterns ) |
| 1. TITLE-ABS-KEY ( clusters  OR  patterns  OR  behaviour  OR  habits  OR  clustering  OR  co-existing  OR  co-occuring  OR  synergistic ) |
| 1. TITLE-ABS-KEY ( behavioural  AND  patterns ) |
| 1. TITLE-ABS-KEY ( behaviour  AND  patterns ) |
| 1. TITLE-ABS-KEY ( lifestyle  AND  patterns ) |
| 1. TITLE-ABS-KEY ( cluster  AND  analysis ) |
| 1. TITLE-ABS-KEY ( health  AND  behaviour ) |
| 1. #14  AND  #15  AND  #16  AND  #17  AND  #18  AND  #19  AND  #20 |
| 1. TITLE-ABS-KEY ( dietary  AND  behaviours ) |
| 1. TITLE-ABS-KEY ( diet  OR  nutrition  OR  snacks  OR  snack*  OR  foods  OR  eating ) |
| 1. TITLE-ABS-KEY ( snacking  OR  fruits  OR  vegetables  OR  fruit*  OR  vegetable*  OR  energy-dense ) |
| 1. TITLE-ABS-KEY ( soft  AND  drink ) |
| 1. TITLE-ABS-KEY ( unhealthy  AND  foods ) |
| 1. TITLE-ABS-KEY ( energy  AND  intake ) |
| 1. TITLE-ABS-KEY ( sweet  AND  drinks ) |
| 1. TITLE-ABS-KEY ( unhealthy  AND  snacks ) |
| 1. TITLE-ABS-KEY ( sugar  AND  sweetened  AND  beverag* ) |
| 1. TITLE-ABS-KEY ( energy-dense  AND  snack* ) |
| 1. TITLE-ABS-KEY ( sweet  AND  snacks ) |
| 1. TITLE-ABS-KEY ( savoury  AND  snacks ) |
| 1. TITLE-ABS-KEY ( junk  AND  food ) |
| 1. #22  AND  #23  AND  #24  AND  #25  AND  #26  AND  #27  AND  #28  AND  #29  AND  #30  AND  #31  AND  #32  AND  #33  AND  #34 |
| 1. TITLE-ABS-KEY ( physical  AND  activity ) |
| 1. TITLE-ABS-KEY ( exercise  OR  physical*  OR  active*  OR  activit*  OR  movement  OR  accelerometer  OR  sport* ) |
| 1. TITLE-ABS-KEY ( physical  AND  fitness ) |
| 1. TITLE-ABS-KEY ( motor  AND  activity ) |
| 1. TITLE-ABS-KEY ( motor  AND  active ) |
| 1. TITLE-ABS-KEY ( physically  AND  active ) |
| 1. TITLE-ABS-KEY ( physical  AND  exercise ) |
| 1. TITLE-ABS-KEY ( movement  AND  behaviours ) |
| 1. TITLE-ABS-KEY ( physical  AND  effort ) |
| 1. TITLE-ABS-KEY ( physical  AND  exertion ) |
| 1. TITLE-ABS-KEY ( bicycle*  OR  swim*  OR  walk*  OR  run*  OR  jog*  OR  play  OR  playthings  OR  outdoor*  OR  activ*  OR  playground* ) |
| 1. #36  AND  #37  AND  #38  AND  #39  AND  #40  AND  #41  AND  #42  AND  #43  AND  #44  AND  #45  AND  #46 |
| 1. TITLE-ABS-KEY ( sedentary  AND  behaviour ) |
| 1. TITLE-ABS-KEY ( sedentariness  OR  sedentary  OR  tv  OR  inactiv*  OR  sitting  OR  screentime  OR  television  OR  computers ) |
| 1. TITLE-ABS-KEY ( sedentary  AND  lifestyle ) |
| 1. TITLE-ABS-KEY ( television  AND  viewing ) |
| 1. TITLE-ABS-KEY ( screen  AND  time ) |
| 1. TITLE-ABS-KEY ( recreational  AND  screen  AND  time ) |
| 1. TITLE-ABS-KEY ( tv  AND  viewing ) |
| 1. TITLE-ABS-KEY ( electronic  AND  gam* ) |
| 1. TITLE-ABS-KEY ( video  AND  gam* ) |
| 1. TITLE-ABS-KEY ( videogam*  OR  software  OR  internet  OR  smartphone*  OR  ipad  OR  apps  OR  app ) |
| 1. TITLE-ABS-KEY ( social  AND  media ) |
| 1. TITLE-ABS-KEY ( mobile  AND  applications ) |
| 1. #48  AND  #2  AND  #3  AND  #4  AND  #5  AND  #6  AND  #7  AND  #8  AND  #9  AND  #10  AND  #11  AND  #59 |
| 1. ( #22  AND  #23  AND  #24  AND  #25  AND  #26  AND  #27  AND  #28  AND  #29  AND  #30  AND  #31  AND  #32   AND  #33  AND  #34 )  AND  ( #36  AND  #37  AND  #38  AND  #39  AND  #40  AND  #41  AND  #42  AND  #43  AND  #44  AND  #45  AND  #46 )  AND  ( #48  AND  #2  AND  #3  AND  #4  AND  #5  AND  #6  AND  #7  AND  #8  AND  #9  AND  #10  AND  #11  AND  #59 ) |
| 1. ( #4  OR  #9  OR  #12 )  AND  ( #14  AND  #15  AND  #16  AND  #17  AND  #18  AND  #19  AND  #20 )  AND   ( ( #22  AND  #23  AND  #24  AND  #25  AND  #26  AND  #27  AND  #28  AND  #29  AND  #30  AND  #31  AND  #32  AND  #33  AND  #34 )  AND  ( #36  AND  #37  AND  #38  AND  #39  AND  #40  AND  #41  AND  #42  AND  #43  AND  #44  AND  #45  AND  #46 )  AND  ( #48  AND  #2  AND  #3  AND  #4  AND  #5  AND  #6  AND  #7  AND  #8  AND  #9  AND  #10  AND  #11  AND  #59 ) ) |
