# Supplementary material for: Clustering of diet, physical activity and sedentary behaviour and related physical and mental health outcomes: a systematic review
Source: BMC Public Health. 2023 Aug 18;23:1572. doi: 10.1186/s12889-023-16372-6 (PMC10436445; doi:10.1186/s12889-023-16372-6)
Supplement: Supplementary file 2 — Additional file 2. [file 12889_2023_16372_MOESM2_ESM.docx]

**Additional file 2: Brief summary of study characteristics**

| First author | Year | Location | Study design | Age range (years) | Mean age (years) | Sample size | Behaviour assessed  (measure/outcome) | | | Method used for cluster identification | Number of patterns identified | Sociodemographic characteristics | | | Prevalence | Outcomes of interest | | |
| --- | --- | --- | --- | --- | --- | --- | --- | --- | --- | --- | --- | --- | --- | --- | --- | --- | --- | --- |
|  |  |  |  |  |  |  | PA | SB | Diet |  |  | Sex | SES | Age |  | Adiposity | Cardiometabolic health/fitness | Mental health |
| Children (n=13) | | | | | | | | | | | | | | | | | | |
| Bell | 2016 | Australia | CS | 9–11 | 10.6 | 4637 | Questionnaire/  PAR | Questionnaire/STR | Questionnaire/FVR and DFI | Descriptive | 6 | + | + | + | + | NA | NA | NA |
| Bel-Serrat | 2013 | Europe | CS | 6–9 | 7.7 | 2846 | Questionnaire/  PA | Questionnaire/  TV viewing | Questionnaire/  FV and SSB | CA | 5 | + | NA | + | NA | NA | + | NA |
| Bel-Serrat | 2019 | Multi-Centre | CS | 6–9 | NA | 63215 | Questionnaire/  VPA | Questionnaire/  ST | FFQ/  FV and CSD | CA | 7 | + | NA | NA | + | + | NA | NA |
| Cameron | 2011 | Australia | CS | 5–12 | NA | 352 | Device/  MVPAR | Questionnaire/  STR | Questionnaire/FVR and EDFR | CA | 5 | NA | NA | + | + | NA | NA | NA |
| Drenowatz | 2012 | US | CS | 10–11 | 10.6 | 210 | Questionnaire/  MVPAR | Questionnaire/  STR | FFQ/  HEIR | Descriptive | 4 | NA | NA | NA | NA | NA | + | NA |
| Elsenburg | 2014 | UK | CS | 9–10 | 10.3 | 1472 | Device/  MVPAR | Questionnaire/  STR | Diet-diary/  FVR and MARR | O/E | 3 | NA | NA | NA | + | NA | NA | NA |
| Gubbels | 2011 | Netherlands | PC | 5–8 | NA | 2074 | Questionnaire/  PA | Questionnaire/  TV viewing and computer use | FFQ/  consumption of diverse foods | PCA | 4 | + | + | + | NA | + | NA | NA |
| Kunin-Batson | 2015 | US | CS | 5–10 | 6.6 | 421 | Device/  MVPAR | Questionnaire/  STR | 24h dietary recall/  FVR and SSBR | Descriptive | 3 | NA | NA | NA | + | NA | NA | NA |
| López-Gil | 2020 | Spain | CS | 6–13 | NA | 353 | Questionnaire/  MVPA and sport activities | Questionnaire/  ST | Questionnaire/  MDQI | CA | 3 | + | NA | + | + | NA | NA | NA |
| Pereira | 2015 | Portugal | CS | 9–11 | NA | 686 | Device/  MVPA | Questionnaire/  ST | FFQ/  FV and SSB | LCA | 3 | NA | NA | NA | + | NA | NA | NA |
| Rodenburg | 2013 | Netherlands | CS | 8–11 | 8.2 | 1480 | Questionnaire/  activity preferences | Questionnaire/  activity preferences | Questionnaire/  food preferences | PCA | 3 | + | NA | + | + | NA | NA | NA |
| Santaliestra-Pasías | 2015 | Europe | CS | 2-9 | NA | 11,674 | Questionnaire/  sport activities | Questionnaire/  SB | FFQ/  FV and SSB | CA | 6 | + | + | + | + | + | NA | NA |
| Yang-Huang | 2020 | Netherlands | CS | 6 | 6 | 4059 | Questionnaire/  sport activities | Questionnaire/  ST | Questionnaire/  SSB and SN | CA | 3 | + | + | NA | + | NA | NA | NA |
| Adolescents (n=31) | | | | | | | | | | | | | | | | | | |
| Berlin | 2017 | US | CS | 13.21 - 15.38 | 14.07 | 9304 | Questionnaire/  sport activities and VPA | Questionnaire/  TV viewing, computer and internet use | Questionnaire/  consumption of diverse foods | LPA | 3 | + | + | NA | + | NA | NA | NA |
| Cuenca-García | 2013 | Europe | CS | 12.5 – 17.5 | 14.8 | 2084 | Questionnaire/  MVPA | Questionnaire/  ST and SBHW | 24h dietary recall/  DQI | CA | 5 | + | NA | + | + | + | + | NA |
| Cureau | 2018 | Brazil | CS | 12 – 17 | NA | 62,063 | Questionnaire/  MVPA | Questionnaire/  ST | 24h dietary recall/  fiber intake | Descriptive | 1 | + | NA | NA | + | + | NA | NA |
| Dantas | 2018 | Brazil | CS | 12–18 | NA | 578 | Questionnaire/  PA | Questionnaire/  ST | Questionnaire/  FV and SSB | CA | 6 | + | + | + | + | + | NA | NA |
| de Mello | 2021 | Brazil | CS | 13–14 | 13 | 812 | Questionnaire/  PA | Questionnaire/  ST | Questionnaire/  FV and SSS | LCA | 2 | + | NA | NA | + | + | NA | NA |
| de Mello | 2022 | Brazil | CS | 11 – 19 | NA | 16,336 | Questionnaire/  PA | Questionnaire/  SB | Questionnaire/  FV and SSS | CA | 4 | + | + | + | + | NA | NA | NA |
| Foltz | 2011 | US | CS | 12–19 | NA | 4414 | Questionnaire/  MPAR or VPAR | Questionnaire/  STR | Multiple pass recall methods/  FVR and SSBR | Descriptive | 1 | + | + | NA | + | NA | NA | NA |
| Hardy | 2012 | Australia | CS | 10 – 16 | Grade 6 (11.6)  Grade 8 (13.6)  Grade 10  (15.6) | 1568 | Questionnaire/  MVPA | Questionnaire/  ST | FFQ/  FV, SN, CSD | O/E | 7 | + | + | NA | + | NA | NA | NA |
| Hartz | 2018 | US | CS | 12–19 | 15.4 | 1233 | Device/  VPA, MPA and MVPA | Device/  SB  Questionnaire/  TV viewing and computer use | 24h dietary recall/  DQI | LCA | 3 | + | NA | + | + | NA | + | NA |
| Ianotti and Wang | 2013 | US | CS | 11 – 16 | 13.9 | 9206 | Questionnaire/  PA | Questionnaire/  TV viewing and computer use | FFQ/  FV, SN, CSD, chips and French fries | LCA | 3 | + | + | + | + | NA | NA | NA |
| Idelson | 2013 | Italy | CS | 14 –17 | 16.3 | 478 | Questionnaire/  MVPAR | Questionnaire/  TV viewing R | FFQ/  FVR, BF R, and milk/yogurt R | Descriptive | 1 | + | NA | NA | + | NA | NA | NA |
| Kerkadi | 2021 | Algeria | CS | 11–16 | NA | 4189 | Questionnaire/  PA | Questionnaire/  SB | Questionnaire/  FV | O/E | 1 | NA | NA | NA | + | NA | NA | NA |
| Khan | 2019 | Bangladesh | CS | 11–17 | 14.2 | 2978 | Questionnaire/  PA | Questionnaire/  SB | Questionnaire/  FV | O/E | 1 | + | NA | NA | + | NA | NA | NA |
| Long | 2021 | Vietnam | CS | 13–17 | NA | 7541 | Questionnaire/  MVPA | Questionnaire/  SB | Questionnaire/  FV, CSD and FF | LCA | 3 | + | NA | NA | + | NA | NA | NA |
| Magalhães | 2022 | Brazil | CS | 10–16 | 12.9 | 1159 | Questionnaire/  PA | Questionnaire/  ST | Questionnaire/  FV and UPF with excess salt and sugar | CA | 5 | + | NA | NA | + | NA | + | NA |
| Maia | 2018 | Brazil | CS | 14–15 | NA | 109,104 | Questionnaire/  PA | Questionnaire/  TV viewing and SITT | Questionnaire/  HF and UHF | CA | 2 | + | + | + | + | NA | NA | NA |
| Mandic | 2017 | New Zealand | CS | 13–18 | 15.3 | 1300 | Questionnaire/  PAR, MVPA, sport activities and active commuting | Questionnaire/  STR, ST, TV viewing and computer use | Questionnaire/  FVR, FV, SN, CSD, FF and BF | CA | 6 | + | + | + | + | NA | NA | NA |
| Matias | 2017 | Brazil | CS | 11–19 | 14.3 | 102,072 | Questionnaire/  PA | Questionnaire/  SB | Questionnaire/  HF and UHF | CA | 3 | + | + | + | + | NA | NA | NA |
| Moreira | 2018 | Europe and Brazil | CS | 12.5–19 | NA | 3025 | Questionnaire/  MVPA | Questionnaire/  TV viewing | 24h dietary recall/  FV  FFQ/  SSB | CA | 5 | + | + | + | + | + | NA | NA |
| Niermann | 2018 | Germany | CS | 12 –14 | 14.02 | 189 | Questionnaire/  MVPA | Questionnaire/  ST | FFQ/  HF and SW | CA | 3 | + | + | NA | + | NA | NA | NA |
| Nunes | 2016 | Brazil | CS | 14–19 | 16.1 | 916 | Questionnaire/  PA | Questionnaire/  ST | Questionnaire/  UHF | O/E | 1 | + | + | + | + | NA | NA | NA |
| Ottevaere | 2011 | Europe | CS | 12.5–17.5 | Younger age  13.8  Older age  16.0 (males) and 15.9 (females) | 2084 | Questionnaire/  MVPA | Questionnaire/  ST | 24h dietary recall/  DQI | CA | 5 | + | + | + | + | NA | NA | NA |
| Sanchez | 2007 | US | CS | 11–15 | 12.7 | 878 | Device/  PAR | Questionnaire/  TV viewing R | 24h dietary recall/  FVR and FR | Descriptive | 3 | + | NA | + | + | NA | NA | NA |
| Silva | 2014 | Brazil | CS | 15–19 | NA | 6529 | Questionnaire/  MVPA | Questionnaire/  ST | Questionnaire/  FV | O/E | 1 | + | NA | NA | + | NA | NA | NA |
| Spengler | 2012 | Germany | CS | 11–17 | 13.7 | 1643 | Questionnaire/  PA | Questionnaire/  Media use | FFQ/  DQI | CA | 4 | + | + | + | + | NA | NA | NA |
| Spengler | 2014 | Germany | PC | 11–24 | T1: 13.5  T2: 20.2 | 1642 | Questionnaire/  PA | Questionnaire/  Media use | FFQ/  DQI | CA | 4 | NA | NA | NA | + | + | NA | NA |
| Teh | 2019 | Malaysia | CS | 16–17 | NA | 2538 | Questionnaire/  PA | Questionnaire/  SB | Questionnaire/  FV | O/E | 1 | + | NA | NA | + | NA | NA | NA |
| Uddin | 2020 | Multi-Centre | CS | 11–17 | 14.4 | 304,779 | Questionnaire/  PA | Questionnaire/  SITT | Questionnaire/  FV | O/E | 1 | + | NA | NA | + | NA | NA | NA |
| Van der Sluis | 2010 | Norway | CS | 11–13 | 11.8 | 884 | Questionnaire/  sport activities | Questionnaire/  TV viewing | Questionnaire/  FV, SN and CSD | CA | 4 | NA | NA | NA | NA | + | NA | NA |
| Veloso | 2012 | Portugal | CS | 10–21 | 14.8 | 3069 | Questionnaire/  MVPA | Questionnaire/  ST | Questionnaire/  FV, CSD and SN | CA | 3 | + | NA | + | + | + | NA | + |
| Wadolowska | 2018 | Poland | CS | 11–13 | NA | 1549 | Questionnaire/  PA | Questionnaire/  ST | FFQ/  consumption of diverse foods | CA | 3 | + | + | + | + | + | NA | NA |
| Children and Adolescents (n=7) | | | | | | | | | | | | | | | | | | |
| Khoshhali | 2021 | Iran | CS | 7–18 | 12.28 | 14,270 | Questionnaire/  PA | Questionnaire/  ST | Questionnaire/  consumption of diverse foods | LCA | 3 | + | + | NA | + | + | NA | NA |
| Leech | 2014 | Australia | CS | 5–6  10–12 | 5.8  11.2 | 362  610 | Device/  MVPA | Device/  SB  Questionnaire/  TV viewing | FFQ/  FV and EDF | CA | 3 | + | + | + | + | NA | NA | NA |
| Leech | 2015 | Australia | PC | 5–6  10–12 | 5.8  11.2 | 87  123 | Device/  MVPA | Device/  SB  Questionnaire/  TV viewing | FFQ/  FV and EDF | CA | 3 | + | NA | + | + | + | NA | NA |
| Mayne | 2020 | US | CS | 5–19 | NA | 7714 | Questionnaire/  PA (children)  Questionnaire/  MVPA (adolescents) | Questionnaire/  ST | 24h dietary recall/  DQI and FF | Descriptive | 2 | NA | NA | + | + | NA | NA | NA |
| Sánchez-Oliva | 2018 | Spain | PC | 8–18 | 12.45 | 1634 | Device/  MVPA | Device/  SB  Questionnaire/  ST | Questionnaire/  MDQI | CA | 4 | + | + | + | + | + | NA | NA |
| Schmiege | 2016 | US | CS | 3–18 | 9.3 | 971 | Questionnaire/  PA | Questionnaire/  ST | Questionnaire/  FV, SSB, SN, BF, and restaurants | LCA | 4 | + | NA | + | + | NA | NA | NA |
| Schroder | 2017 | Spain | CS | 5–18 | NA | 1614 | Questionnaire/  PA | Questionnaire/  ST | Questionnaire/  BF and MF | Descriptive | 3 | + | + | + | + | + | NA | NA |
| Adolescents and young Adults (n=1) | | | | | | | | | | | | | | | | | | |
| Watts | 2015 | UK | CS | 16–24 | NA | 1,214 | Questionnaire/  MPA | Questionnaire/  SITT | FFQ/  FV | LCA | 1 | NA | NA | NA | + | NA | NA | NA |
| Young Adults (n=1) | | | | | | | | | | | | | | | | | | |
| Al-Nakeeb | 2015 | Qatar | CS | 18–25 | 22.0 (males)  20.6 (females) | 320 | Questionnaire/  PA | Questionnaire/  TV viewing and computer use | Questionnaire/  consumption of diverse foods | CA | 3 | + | NA | + | + | NA | NA | NA |

Abbreviations: PA, physical activity; SB, sedentary behaviours; SES, socioeconomic status; CS, cross-sectional; PAR, physical activity recommendations; STR, screen-time recommendations; FVR, fruits and vegetables intake recommendations; DFI, discretionary food intake; Descriptive, co-prevalence of behaviours ;+ , assessed ;NA, not assessed; TV, television; FV, fruits and vegetables; SSB, sugar-sweetened beverages; CA, cluster analysis ;VPA, vigorous physical activity; ST, screen-time; FFQ, food frequency questionnaire; CSD, carbonated soft drink; MVPAR, moderate to vigorous physical activity recommendations; EDFR, energy-dense food recommendations; HEI, healthy eating index recommendations; MARR, mean adequacy ratio recommendations; O/E, Observed over expected ratio; PC, prospective cohort; PCA, principal component analysis; SSBR, sugar-sweetened beverages recommendations; MVPA, moderate to vigorous physical activity ;MDQI, mediterranean diet quality index; LCA, latent class analysis; SN, snack; LPA, latent profile analysis; SBHW, sedentary behaviour devoted to homework; DQI, diet quality index; SSS, sugar, salty snacks and soda; MPAR, moderate physical activity recommendations; VPAR, vigorous physical activity recommendations; MPA, moderate physical activity; R, recommendations; BF, breakfast; FF, fast foods; UPF, ultra-processed food; SITT, sitting time; HF, healthy foods, UHF, unhealthy foods; SW, sweets; FR, fat recommendations; T1, time at baseline; T2, time at follow-up; EDF, energy-dense food; MF, meals frequency.
