# Supplementary material for: Clustering of diet, physical activity and sedentary behaviour and related physical and mental health outcomes: a systematic review
Source: BMC Public Health. 2023 Aug 18;23:1572. doi: 10.1186/s12889-023-16372-6 (PMC10436445; doi:10.1186/s12889-023-16372-6)
Supplement: Supplementary file 3 — Additional file 3. [file 12889_2023_16372_MOESM3_ESM.docx]

**Additional file 3: Summary of lifestyle patterns identified**

| Lifestyle behaviours assessed | | | | | | | | | |
| --- | --- | --- | --- | --- | --- | --- | --- | --- | --- |
| Type of pattern | **Diet** | | | **Physical activity** | | | **Sedentary behaviours** | | |
|  | **High** | **Low** | **Moderate** | **High** | **Low** | **Moderate** | **High** | **Low** | **Moderate** |
| Healthy  (n=28) | HF | SW |  | MVPA |  |  |  | ST |  |
|  | HF | UHF |  | PA |  |  |  | SB |  |
|  | FV | DISF |  | PA |  |  |  | ST |  |
|  | FV | ED |  | PA |  |  |  | ST |  |
|  | FV | FF |  | PA |  |  |  | ST |  |
|  |  | SSB  SN |  | PA |  |  |  | ST |  |
|  |  | SSB |  | MVPA |  |  |  | ST |  |
|  | FV |  |  | MVPA |  |  |  | ST |  |
|  | DQ |  |  | MVPA |  |  |  |  |  |
|  |  |  |  | MVPA |  |  |  |  |  |
|  | FV | ED |  |  |  |  |  | ST |  |
|  | BF  DP  FV |  |  | MVPA |  |  |  | TV |  |
|  | DQ |  |  | MVPA |  |  |  | ST |  |
|  |  |  |  | PA |  |  |  |  |  |
|  | FV  BF | SN  SSB |  | PA |  |  |  | ST |  |
|  | FV | SW  CSD |  | PA |  |  |  | SB |  |
|  | MED |  |  | MVPA |  |  |  | ST  SB |  |
|  | FV | SN  CSD |  | PA |  |  |  | SB |  |
|  | HF  BF |  |  | VPA |  |  |  | ST |  |
|  | HF |  |  |  |  |  |  |  |  |
|  | HF |  |  | PA |  |  |  |  |  |
|  | FV | CSD |  | VPA |  |  |  | ST |  |
|  | FV | CSD |  |  |  |  |  |  |  |
|  |  |  |  | VPA |  |  |  |  |  |
|  | DQ |  |  | MVPA |  |  |  | SB |  |
|  | FV |  |  | PA |  |  |  |  |  |
|  | FV | SSB |  | MVPA |  |  |  | ST |  |
|  |  | ED |  | MVPA |  |  |  | SB  TV |  |
| Unhealthy  (n=46) |  | FV |  |  | MPA |  | SITT |  |  |
|  | MAR | FV |  |  | MVPA |  | ST |  |  |
|  | SW | HF |  |  | MVPA |  | ST |  |  |
|  |  | HEI |  |  | PA |  | ST |  |  |
|  | FF |  |  |  | PA |  | ST |  |  |
|  | ED | FV |  |  |  |  | TV |  |  |
|  |  |  |  |  | MVPA |  | SB |  |  |
|  | UHF | HF |  |  | PA |  | SB |  |  |
|  | UHF |  |  |  | PA |  | ST |  |  |
|  | SSB | FV |  |  | MVPA |  | ST |  |  |
|  |  | DQ |  |  | PA |  | MU |  |  |
|  | UHF | HF |  |  | MPA |  | TV |  |  |
|  |  |  |  |  | PA |  | ST |  |  |
|  | SN  CSD | FV |  |  | MVPA |  | ST |  |  |
|  | CSD | FV |  |  | MVPA |  | ST |  |  |
|  | SN | FV |  |  | MVPA |  | ST |  |  |
|  | SN  CSD |  |  |  | MVPA |  | ST |  |  |
|  | SN |  |  |  | MVPA |  | ST |  |  |
|  | CSD |  |  |  | MVPA |  | ST |  |  |
|  |  | FV |  |  | MVPA |  |  |  |  |
|  | ED |  |  |  |  |  | ST |  |  |
|  |  | DQ |  |  | PA |  | MU |  |  |
|  | UHF | HF |  |  |  |  |  |  |  |
|  | SSB |  |  |  |  |  | TVCOM |  |  |
|  | JF | FV |  |  | PA |  | ST |  |  |
|  | F | FV |  |  | MVPA |  | TV |  |  |
|  | ED |  |  |  |  |  | TV |  |  |
|  |  |  |  |  | MVPA |  | SB |  |  |
|  |  | BF  MF |  |  | PA |  | ST |  |  |
|  |  | BF |  |  | PA |  | ST |  |  |
|  |  | MF |  |  | PA |  | ST |  |  |
|  |  | FIB |  |  | MVPA |  | ST |  |  |
|  | CSD  SN | FV |  |  | PA |  | SB |  |  |
|  | UHF | BF |  |  |  |  | ST |  |  |
|  | UHF |  |  |  |  |  | TV |  |  |
|  |  |  |  |  | VPA |  | ST |  |  |
|  | CSD |  |  |  | VPA |  | ST |  |  |
|  |  | DQ |  |  | MVPA |  | SB |  |  |
|  | SSS | FV |  |  | PA |  | ST |  |  |
|  | UHF | FV |  |  | PA |  | SB |  |  |
|  |  | FV  DP |  |  |  |  |  |  |  |
|  |  | FV |  |  | PA |  | SITT |  |  |
|  |  | DQ |  |  | MVPA |  | ST |  |  |
|  |  | FV |  |  | PA |  | SB |  |  |
|  | UHF |  |  |  | PA |  | SB |  |  |
|  |  | FV |  |  | MVPA |  | ST |  |  |
| Mixed (n=98) | FV  MAR |  |  |  | MVPA |  | ST |  |  |
|  |  | MAR  FV |  |  | MVPA |  | ST |  |  |
|  | HF | SW |  |  | MVPA |  |  | ST |  |
|  |  | FV |  |  | PA |  | SB |  |  |
|  |  | HF  UHF |  |  | PA |  |  | SITT |  |
|  | HF  UHF |  |  | PA |  |  | SITT |  |  |
|  |  | HF  UHF |  |  | PA |  |  | SB |  |
|  | FV  SSB |  |  |  | MVPA |  | ST |  |  |
|  | FV | SSB |  |  | MVPA |  | ST |  |  |
|  | ED |  | FV |  |  | PA | ST |  |  |
|  |  | FV  ED |  |  | PA |  |  |  | ST |
|  |  | FV | FF |  | PA |  |  |  | ST |
|  | FF |  | FV |  |  | PA | ST |  |  |
|  |  | FV |  | PA |  |  | ST |  |  |
|  |  |  | FV |  | PA |  |  | ST |  |
|  |  |  | DQ | PA |  |  |  |  | MU |
|  | DQ |  |  |  | PA |  |  | MU |  |
|  |  | DQ |  |  | PA |  |  | MU |  |
|  | DQ |  |  | MVPA |  |  | ST |  |  |
|  |  | DQ |  |  | MVPA |  |  | ST |  |
|  |  |  | HF  UHF | MPA |  |  |  | TVCOM |  |
|  | HF | UHF |  |  |  | MPA | TVCOM |  |  |
|  | SN  SSB |  |  | PA |  |  |  |  |  |
|  |  |  | DQ |  | MVPA |  | SB |  |  |
|  |  | DQ |  | MVPA |  |  |  |  |  |
|  | FV | ED |  | MVPA |  |  | ST |  |  |
|  |  |  | DQ | PA |  |  |  |  | MU |
|  | DQ |  |  |  | PA |  |  | MU |  |
|  |  | DQ |  |  | PA |  |  | MU |  |
|  | DQ |  |  |  | MVPA |  | ST |  |  |
|  |  | DQ |  | MVPA |  |  |  | ST |  |
|  |  | JF  FV |  |  | PA |  |  | ST |  |
|  | SN  SSB  FV  BF |  |  | PA |  |  | ST |  |  |
|  | F  FV |  |  |  | MVPA |  | TV |  |  |
|  |  | F  FV |  |  | MVPA |  | TV |  |  |
|  | SW  CSD | FV |  | PA |  |  | ST |  |  |
|  |  | FV | SW  CSD |  | PA |  |  | SB | TV |
|  |  | MED |  |  | MVPA |  | SB | ST |  |
|  |  |  | MED |  |  | MVPA | SB |  | ST |
|  | MED |  |  |  | MVPA |  |  | ST | SB |
|  |  |  | MED |  |  | MVPA | ST |  | SB |
|  |  | MED |  |  | MVPA |  |  |  | ST  SB |
|  |  | SN  CSD | FV | PA |  |  |  |  | SB |
|  |  | FV  SN  CSD |  |  |  | PA | SB |  |  |
|  |  | HF  UHF  BF |  |  | VPA |  |  |  |  |
|  |  | FV  SSB |  |  | MVPA |  | TV |  |  |
|  | SSB | FV |  |  | MVPA |  |  |  | TV |
|  | SSB |  | FV | MVPA |  |  | TV |  |  |
|  |  | FV  SSB |  |  | MVPA |  | TV |  |  |
|  | SSB | FV |  |  | MVPA |  |  | TV |  |
|  | FV  SSB |  |  | MVPA |  |  |  | TV |  |
|  | UHF  HF |  |  |  |  |  |  |  |  |
|  |  | FV  CSD |  |  | VPA |  |  | ST |  |
|  | FV  CSD |  |  |  |  |  |  |  |  |
|  | FV | CSD |  |  | VPA |  | ST |  |  |
|  | CSD |  |  | VPA |  |  | ST |  |  |
|  | FV | CSD |  | VPA |  |  | ST |  |  |
|  | FV  CSD |  |  | VPA |  |  | ST |  |  |
|  | DQ |  |  | MVPA |  |  |  | ST | SBHW |
|  | DQ |  |  |  |  | MVPA | SBHW |  | ST |
|  |  |  | DQ |  | MVPA |  |  |  | ST  SBHW |
|  |  | DQ |  |  |  | MVPA | ST |  | SBHW |
|  |  | DQ |  | MVPA |  |  |  |  | ST  SBHW |
|  | DQ |  |  |  | MVPA |  | SB |  |  |
|  | DQ |  |  | MVPA |  |  | SB |  |  |
|  |  | FV  SSB |  | PA |  |  |  | TV |  |
|  |  | FV  SSB |  |  | PA |  | TV |  |  |
|  |  | FV  SSB |  | PA |  |  | TV |  |  |
|  | SSB | FV |  |  | PA |  |  | TV |  |
|  | FV | SSB |  |  | PA |  |  | TV |  |
|  |  | FV  SSB |  |  | PA |  |  | TV |  |
|  | SSS | FV |  | PA |  |  | ST |  |  |
|  |  | FV  UHF |  |  | PA |  |  | ST |  |
|  |  | UHF |  |  | PA |  |  | ST |  |
|  | FV |  |  | PA |  |  |  |  | SB |
|  |  |  | FV |  | PA |  |  |  | SB |
|  |  | FV  UHF |  |  | PA |  |  |  | SB |
|  | SW  SSB  SN  FF | FV  DP |  | PA |  |  |  |  |  |
|  | FF  SW  SSB  SN | BF  FV  DP |  | PA |  |  |  |  |  |
|  | DQ |  |  |  | MVPA |  |  | SB |  |
|  |  | DQ |  |  | MVPA |  |  | SB |  |
|  | FV |  |  |  | PA |  | ST |  |  |
|  |  | FV  SSB |  |  | PA |  | SB |  |  |
|  |  |  | MED | MVPA |  |  |  | ST  SB |  |
|  |  | FV  SSB |  | PA |  |  |  | SB |  |
|  | MED |  |  |  | MVPA |  | SB | ST |  |
|  |  | MED |  |  |  | MVPA | ST |  | SB |
|  |  | FV  SSB |  | PA |  |  | SB |  |  |
|  | SSB | FV |  |  | PA |  |  | SB |  |
|  |  | FV  SSB |  |  | PA |  |  | SB |  |
|  | FV | SSB |  |  | PA |  |  | SB |  |
|  |  | FV  SSB |  | MVPA |  |  |  | TV |  |
|  | FV | SSB |  |  | MVPA |  |  | TV |  |
|  |  | FV  SSB |  |  | MVPA |  |  | TV |  |
|  |  |  |  | VPA |  |  | ST |  |  |
|  | FV | SSS |  | PA |  |  | ST |  |  |
|  | FV  SSS |  |  |  | PA |  | ST |  |  |
|  | UHF |  |  | PA |  |  | ST |  |  |

Abbreviations: n, number of patterns found; MVPA, moderate to vigorous physical activity; FV, fruits and vegetables; MAR, mean adequacy ratio; ST, screen time; HF, healthy foods; SW, sweets; PA, physical activity; UHF, unhealthy foods; SB, sedentary behaviours; DISF, discretionary foods; ED, energy dense; FF, fast foods; SSB, sugar sweetened beverages; SN, unhealthy snacks; DQ, diet quality; BF, breakfast; DP, dairy products; TV, television; CSD, carbonated soft drink; MED, Mediterranean diet; VPA, vigorous physical activity; MPA, moderate physical activity; SITT, sitting time; HEI, healthy eating index; MU, media use; TVCOM, television and computer; JF, junk food; F, fat; MF, meal frequency; FIB, fibre; SSS, sugar, salty snacks and soda; SBHW, sedentary behaviours devoted to homework.
